# AQF-Net: Adaptive query modeling and efficient feature fusion for UAV tiny-object detection

**DOI:** 10.1371/journal.pone.0355299

**Published:** 2026-08-13

**Authors:** Yong He, Yifan Tang, Renfeng Xiao, Yufan Pang

**Affiliations:** College of Artificial Intelligence, Changsha University of Science and Technology, Changsha, China; RLBCAU: Rani Lakshmi Bai Central Agricultural University, INDIA

## Abstract

Tiny-object detection in UAV aerial imagery remains challenging due to extremely small object scales, dense distributions, and complex backgrounds. Existing methods often suffer from inefficient query modeling and inadequate multi-scale feature representation, particularly in high-resolution scenarios with substantial variations in target density. To address these challenges, this paper proposes AQF-Net, a unified detection framework built upon the D-FINE architecture. AQF-Net integrates three key components: a Fixed-Query Self-Attention (FQSA) mechanism for efficient global context modeling, a Large-Receptive-Field Enhancement (LREA) module for enhanced multi-scale feature fusion, and an adaptive query modeling strategy for density-aware query allocation. These components are tightly coupled to jointly optimize feature representation and query generation, enabling the model to better adapt to complex UAV scenarios. Extensive experiments are conducted on the CODrone, VisDrone2019, and a self-constructed photovoltaic defect dataset (PV-DV). The results demonstrate that AQF-Net consistently outperforms the D-FINE baseline and several state-of-the-art methods in both overall detection accuracy and tiny-object detection capability. Notably, AQF-Net achieves 33.4% AP and 55.0% AP_50_ on the VisDrone2019 validation set, while maintaining a favorable balance between accuracy and computational efficiency.

## Introduction

With superior air mobility and omni-directional viewing angle, UAV aerial images have become an important means to obtain ground information. The significant impact of UAVs stems primarily from their unique aerial perspective, which enables the collection of extensive visual data and provides comprehensive environmental awareness [1]. With the rapid development of smart cities and the emerging low-altitude economy, unmanned aerial vehicles (UAVs) have seen widespread applications in urban traffic surveillance, public safety inspection, and emergency response [2]. Driven by the rapid expansion of the global unmanned aerial vehicle (UAV) market, the field is undergoing a profound transformation from traditional approaches toward more intelligent, lightweight, and multimodal solutions. The explosion of UAV technology has resulted in an increasing demand for effective and high-performance solutions for online object detection on aerial images [3]. However, the arbitrary orientation of objects caused by the overhead view is the main difference between natural images and aerial images, and it complicates the object detection task in two ways [4]. Due to the imaging altitude in UAV aerial photography, tiny objects occupy very few pixels in images, causing key features such as texture and edges to become indistinct. In addition, complex scenes often contain numerous similar textures, which are easily confused with tiny objects. Therefore, developing effective UAV-based models for tiny-object detection is of great importance. In practical UAV-based traffic and urban monitoring scenarios, tiny-object detection is strongly affected by observation geometry and scene density. Variations in flight altitude change the apparent object scale, while oblique camera angles may introduce perspective distortion and partial occlusion. In dense intersections or crowded urban areas, vehicles and pedestrians often appear in compact groups, leading to severe overlap and low inter-object spacing. Moreover, low contrast between vehicles and road surfaces, shadows from buildings or trees, and cluttered roadside textures further increase background interference. These challenges require UAV detectors to preserve fine-grained local details, capture broader contextual information, and adapt query allocation to different scene densities.

In UAV object detection, the YOLO series is widely favored for its single-stage detection architecture, which significantly reduces computational cost and satisfies the real-time requirements of UAV platforms. To improve detection accuracy in UAV aerial imagery, many researchers worldwide have conducted extensive studies on enhancing YOLO-based methods. Liu *et al* optimized the YOLOv3 architecture by connecting ResNet units of the same size and introducing additional convolutional operations in the initial layers, thereby enlarging the receptive field and improving small-object detection performance [5]. MFP-YOLO [6] adopts a multi-channel inverted residual module in combination with the Convolutional Block Attention Module (CBAM), effectively enhancing the discriminative capability and localization accuracy for small objects. UAV-YOLOv8 [7] introduces the BiFormer attention mechanism to optimize the backbone network, designs a feature processing module termed the Focal FasterNet block, and incorporates two detection scales based on this module, significantly improving detection performance and reducing the miss-detection rate of small objects. LD-YOLOv10 [8] employs a lightweight feature extraction structure, RGELAN, and constructs a DR-PAN neck to alleviate the computational burden of feature extraction while capturing weak object features with lower computational cost. Although these improved YOLO-based methods have enhanced UAV small-object detection performance to some extent, the inherently local receptive fields of convolutional neural networks (CNNs) still limit their ability to capture fine-grained details of tiny objects.

DETR, which is built upon the Transformer architecture, overcomes the limitation of the local receptive fields of CNNs by leveraging a global self-attention mechanism [9]. Its end-to-end design, together with the anchor-free formulation and the elimination of non-maximum suppression (NMS), further simplifies the training pipeline and improves generalization capability. In recent years, Transformer-based detection heads derived from DETR have become a mainstream paradigm in object detection. However, the attention mechanism in DETR is computationally expensive, leading to high computational overhead, slow convergence, and the loss of fine-grained details during deep feature extraction. To address these issues, extensive research has been conducted on DETR variants. Lite-DETR [10] introduces a key-aware deformable attention mechanism and designs an efficient encoder structure that interleaves the updating of high-level and low-level features, thereby enabling more effective multi-scale feature fusion. Its lightweight framework reduces computational cost while maintaining competitive performance; however, Lite-DETR does not explicitly optimize inference speed. DINO-DETR [11] employs contrastive denoising training, a hybrid query selection strategy for anchor initialization, and a look-ahead optimization mechanism for bounding box prediction. These designs alleviate several limitations of DETR-based models, including slow convergence, ambiguous queries, and weak small-object detection performance. However, DINO-DETR is not designed for real-time detection and therefore cannot readily satisfy the requirements of real-time applications. RT-DETR [12] adopts an efficient hybrid encoder and introduces IoU-aware query selection, significantly improving both detection accuracy and inference speed, thus providing a reliable solution for real-time UAV vision tasks. Nevertheless, its ability to detect small objects remains limited. Drone-DETR [13] improves the neck network of RT-DETR by introducing an enhanced dual-path feature fusion attention module and further integrates the P2 layer from the Effective Small Object Detection Network (ESDNet) into the original architecture. This design strengthens the model’s capability to extract and fuse small-object features, thereby improving detection accuracy; however, its performance may still degrade in challenging environments. Overall, although these Transformer-based DETR variants have achieved substantial progress in global modeling and end-to-end detection, they still face several challenges in high-resolution UAV aerial scenarios with densely distributed tiny objects, including the rapid growth of computational complexity with increasing resolution, insufficient multi-scale and fine-grained feature representation, and limited adaptability of query mechanisms. These limitations hinder further improvements in both computational efficiency and tiny-object detection performance.

Recent studies have further explored the DETR framework from the perspectives of training supervision and matching mechanisms. Built upon the RT-DETR architecture, D-FINE [14] strengthens localization supervision and training stability through strategies such as Fine-Grained Distribution Refinement (FDR) and Global Optimal Localization Self-Distillation (GO-LSD), effectively alleviating issues including sparse supervision signals, unstable convergence, and limited localization accuracy during the early stages of training. Although D-FINE has demonstrated strong performance on datasets such as COCO and Objects365, its effectiveness on other datasets remains to be further validated.

To address the above challenges, we propose AQF-Net, a unified framework built upon the D-FINE architecture that integrates adaptive query modeling with efficient multi-scale feature fusion for UAV tiny-object detection. By introducing density-aware query adaptation and enhanced feature interaction into the D-FINE framework, AQF-Net improves both detection accuracy and computational efficiency in high-resolution UAV scenarios. The main contributions of this paper are summarized as follows:

(1) A Fixed-Query Self-Attention (FQSA) mechanism is proposed. By performing attention computation on a fixed number of low-resolution query tokens, FQSA effectively reduces computational complexity under high-resolution settings while preserving global contextual modeling capability. This design removes the quadratic dependency of attention cost on input resolution, making it particularly suitable for high-resolution image processing.
(2) A Large-Receptive-Field Enhancement (LREA) module is designed to address limited receptive fields and insufficient feature fusion in resource-constrained settings. By introducing the Sparse Decomposed Broad Convolution (SDBConv) module and the Bidirectional Selective Aggregation (BSA) module, LREA expands the receptive field through the combination of large-kernel convolution and strip-shaped dilated convolution, thereby enhancing the perception of tiny objects. Meanwhile, it adaptively selects and fuses multi-scale features, thereby further improving robustness in complex backgrounds.
(3) An adaptive query modeling strategy is introduced to improve detection performance, particularly for tiny objects in complex aerial scenes. Built upon the D-FINE Transformer, this strategy incorporates PKIBlock and a dynamic query allocation mechanism, while adopting a lightweight design for the counting-guided module. As a result, it dynamically adjusts the number of queries according to scene complexity and optimizes feature extraction, thereby improving target matching quality and tiny-object detection performance in complex backgrounds.

### Related work

#### Tiny object detection.

Tiny-object detection has long been regarded as one of the most challenging problems in object detection. In UAV aerial scenarios, conventional detectors tend to lose critical information during feature downsampling and semantic aggregation because of the high imaging altitude and top-down perspective. TOD-CNN [15] is designed for tiny object detection in microscopic video data. It adopts a one-stage CNN detection framework together with feature fusion to improve localization and recognition performance; however, both the method and the data distribution are highly dependent on specific scenarios, resulting in limited generalization capability. LE-YOLO [16] introduces a lightweight redesign based on YOLOv8n and enhances the representation of tiny objects in UAV aerial imagery by improving feature extraction and multi-scale fusion, thereby achieving higher detection accuracy with fewer parameters. FSANet [17] proposes a Feature-Aware Alignment Module (FA2M) to learn the spatial transformation offsets between adjacent feature levels during pyramid fusion, thereby alleviating feature misalignment. It also designs a Spatial-Aware Guidance Head (SAGH), which leverages geometric information to perform coarse-to-fine progressive regression, further improving the representation and localization accuracy of tiny objects. SETR-Net [18] introduces a Subtle Context Enhancement (SCE) module to enhance the receptive field and feature representation, thereby avoiding complex multi-scale fusion. It also designs a Saliency Attention Transfer (SAT) mechanism to incorporate temporal recurrence into video saliency prediction. This design improves long-range cross-frame information capture and prediction accuracy, while enhancing the recognition of small-scale or sparse targets in complex scenes. MFFSODNet [19] enhances tiny-object perception while reducing the number of parameters by replacing the large-object prediction head with a tiny-object prediction head. It further introduces a Multi-Branch Multi-Scale Feature Extraction Module (MSFEM) and a Bidirectional Dense Feature Pyramid Network (BDFPN) to enhance fine-grained feature extraction and cross-layer multi-scale fusion, thereby improving tiny-object detection performance on datasets such as VisDrone and UAVDT. However, feature modeling in these methods still relies largely on local convolution operations or fixed-scale structures. As a result, their capabilities for receptive field expansion and multi-scale feature fusion remain limited, making it difficult to simultaneously ensure the discriminability and robustness of tiny objects in complex backgrounds.

To further improve contextual modeling for tiny objects, recent studies have increasingly focused on feature enhancement modules centered on efficient receptive field expansion and multi-scale information fusion. RGCSPELAN [20] enhances gradient flow and feature representation through lightweight cross-stage partial connections. CSSC [21] expands the effective receptive field while maintaining computational efficiency by employing strip and sparse convolution structures. CFBlock [22] approximates the contextual modeling capability of Transformers in a convolutional form, enabling efficient fusion of local and global information. FCM [23] strengthens the interaction between shallow spatial information and deep semantic features through a complementary mapping mechanism, whereas FMB [24] enhances feature representation by integrating high- and low-frequency features at different scales from a frequency-domain perspective. In addition, the InceptionNeXt Block [25] enables more flexible receptive field modeling while preserving computational efficiency through a multi-branch large-kernel decomposition structure. However, these modules are only weakly coupled with end-to-end query matching mechanisms, and their adaptability to scenarios with varying object densities and uneven scale distributions remains limited. Nevertheless, these modules provide a useful design paradigm for improving tiny-object perception under lightweight constraints and also lay the methodological foundation for the proposed LREA module.

Within the DETR family, DQ-DETR [26] introduces a density estimation module to dynamically adjust the number and spatial positions of queries while injecting density-enhanced features into the decoder. This design effectively improves the recall and localization accuracy of tiny objects and achieves considerable performance gains in aerial scenarios. Nevertheless, such methods still have several limitations. First, density maps are typically derived from low-resolution features, which makes it difficult to fully characterize the fine-grained structural information of extremely tiny objects. Second, the coupling between density estimation and query generation increases training complexity, and the efficiency and stability of this paradigm under high-resolution inputs or resource-constrained settings still require further improvement.

### D-FINE Framework

D-FINE (Dynamic Fine-Grained Matching for End-to-End Object Detection) consists of two key components: Fine-Grained Distribution Refinement (FDR) and Global Optimal Localization Self-Distillation (GO-LSD). The core idea of D-FINE is to alleviate the sparse supervision and slow convergence caused by the traditional one-to-one matching strategy in DETR during the early stages of training, while preserving the original end-to-end detection framework. Specifically, D-FINE no longer restricts supervision to a single optimal match. Instead, it dynamically introduces multi-scale and multi-candidate high-quality matching relationships during training according to prediction quality, thereby providing denser and more stable gradient feedback for decoder queries and significantly improving both convergence speed and detection accuracy.

At present, extensions of D-FINE have mainly focused on specific application scenarios, and this line of research is still at an early stage. SAR-D-FINE [27] is designed for detecting small and densely distributed ships in synthetic aperture radar (SAR) imagery. Built upon the D-FINE framework, it introduces a context-aware feature extraction strategy and enhances multi-scale feature fusion, thereby improving the capture of contextual information and the representation of small objects. As a result, the model achieves favorable performance in dense ship detection tasks. However, this method still relies heavily on the characteristics of SAR imagery and the distribution of specific datasets, which may limit its generalization capability in more complex or diverse remote sensing scenarios. IW-D-FINE [28] is developed for ocean internal wave detection in SAR images. Based on the D-FINE architecture, it adopts an anchor-free one-stage detection framework and incorporates several improved modules, including the SimAM attention mechanism, the MultiScalePCA feature fusion module, and a MetaFormer-based encoder structure. These components enhance multi-scale feature extraction and achieve a better balance between local and global feature modeling. Experimental results show that IW-D-FINE attains high detection accuracy while maintaining real-time inference efficiency. HF-D-FINE [29] is a D-FINE-based method for tiny-object detection in UAV imagery. It enhances high-resolution feature representation through the HF Hybrid Encoder, performs cross-scale feature fusion using the CAF module, and introduces the Outer-SNWD loss to improve bounding box regression accuracy for tiny objects. These designs significantly improve detection performance on UAV datasets. However, the method still increases computational complexity to some extent, and its performance gains depend on the trade-off between richer high-resolution feature representations and inference efficiency.

Motivated by these limitations, particularly the suboptimal balance between efficiency and accuracy in high-resolution tiny-object detection, the limited adaptability of existing D-FINE variants across diverse scenarios, and the need for more effective query optimization and multi-scale feature aggregation, we propose AQF-Net. Built upon the D-FINE framework, AQF-Net incorporates a Fixed-Query Self-Attention (FQSA) mechanism and a Large-Receptive-Field Enhancement (LREA) module to improve computational efficiency and multi-scale feature fusion under high-resolution settings. Furthermore, by integrating an adaptive query modeling strategy, AQF-Net optimizes query generation and target matching, thereby improving the detection accuracy and robustness of tiny objects.

## Method

**Fig 1:**
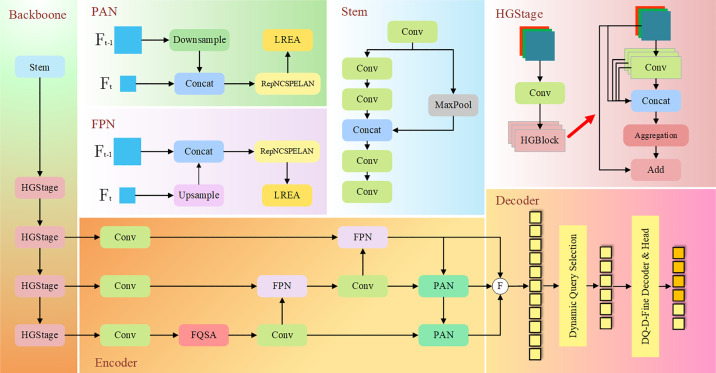
Overall architecture of AQF-Net.

To address the limitations of fixed-query modeling and insufficient multi-scale feature representation in UAV tiny-object detection, we propose AQF-Net, a unified detection framework built upon the D-FINE architecture. The overall architecture is illustrated in Fig 1. AQF-Net is designed under a unified perspective that jointly optimizes adaptive query modeling and efficient feature representation. This design is motivated by the characteristics of UAV aerial scenes, where object scales vary significantly with flight altitude, local details are easily degraded by high-altitude imaging, and target density changes substantially across sparse roads and crowded intersections. Therefore, AQF-Net aims to jointly improve global context modeling, multi-scale feature representation, and density-aware query allocation. Within this framework, three key components are introduced: (1) a Fixed-Query Self-Attention (FQSA) mechanism, (2) a Large-Receptive-Field Enhancement (LREA) module, and (3) an adaptive query modeling strategy. The three components are designed to address different but related challenges in UAV tiny-object detection. FQSA focuses on efficient global context modeling under high-resolution inputs, where conventional self-attention may introduce excessive computational cost. LREA aims to enhance multi-scale and large-receptive-field feature representation, which is important for distinguishing tiny objects from cluttered backgrounds and surrounding structures. The adaptive query modeling strategy further addresses the variation in scene density by adjusting the query budget according to the predicted object distribution. In this way, AQF-Net connects feature enhancement and query allocation within a unified DETR-style detection framework.

These three components are not isolated improvements but are tightly integrated within the AQF-Net framework. Specifically, the efficient attention mechanism improves global context modeling under controlled computational cost, the feature fusion module enhances multi-scale representation, and the adaptive query modeling strategy dynamically adjusts query allocation according to scene characteristics. Together, they enable AQF-Net to achieve a better balance between detection accuracy and computational efficiency. Given an input image, the backbone and neck first extract multi-scale features. These features are refined through the efficient attention and feature fusion modules, and subsequently used to generate adaptive queries. Finally, the dynamically selected queries are fed into the decoder for iterative refinement to produce the final detection results.

### Fixed-Query Self-Attention (FQSA)

Self-attention has shown remarkable capability in modeling long-range dependencies. However, its computational complexity typically grows quadratically with the spatial resolution of input features, resulting in substantial computational overhead in high-resolution dense prediction tasks. Although existing studies have attempted to alleviate this issue through strategies such as window partitioning and sparse connections, these methods often suffer from limited global context modeling or insufficient cross-region information interaction. To address these limitations, we propose a Fixed-Query Self-Attention (FQSA) mechanism. The core idea of FQSA is to generate queries in a fixed low-resolution space and perform attention aggregation with multi-scale keys and values, thereby decoupling the computational complexity of attention from the input resolution and effectively reducing computational cost while preserving a global receptive field.

**Fig 2:**
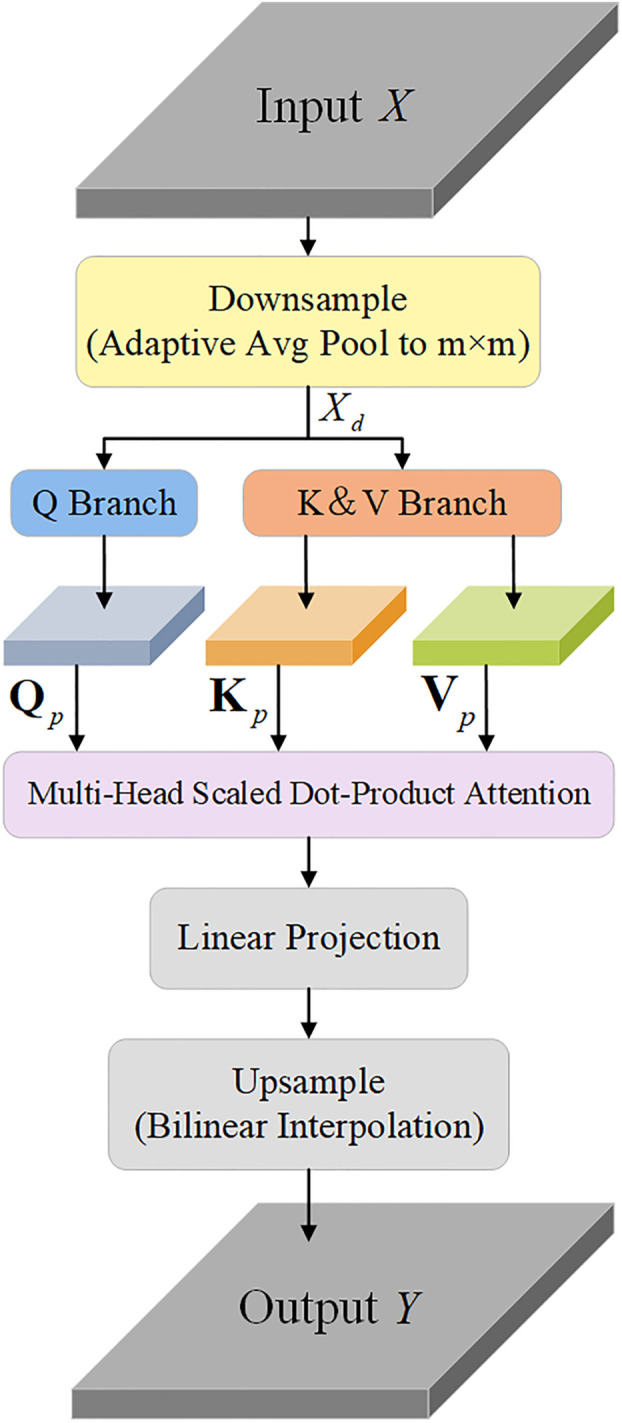
Architecture of FQSA.

The overall architecture of FQSA is illustrated in Fig 2. In this work, the input features $X\in {\mathbb{R}}^{B\times C\times H\times W}$ are downsampled to a low-resolution feature map $X_{d}\in {\mathbb{R}}^{B\times C\times m\times m}$ with a fixed spatial size of m×m using Adaptive Average Pooling. Then, $X_{d}$ is fed into two parallel branches. In the Q branch, $X_{d}$ is linearly transformed to generate the query matrix, and local features are selectively enhanced through lightweight convolution. In the K&V branch, $X_{d}$ is processed through a pyramidal structure to extract multi-scale contextual features, and keys and values are generated via linear transformation. The resulting ${𝐐}_{p}$, ${𝐊}_{p}$, and ${𝐕}_{p}$ are used to compute multi-head scaled dot-product attention in the low-resolution space. Finally, the attention outputs from multiple heads are fused through linear projection and channel alignment, and then restored to the original spatial resolution (H,W) via bilinear interpolation upsampling, producing the final output feature $Y\in {\mathbb{R}}^{B\times C\times H\times W}$.

To overcome the computational bottleneck of standard self-attention under high-resolution settings, FQSA redesigns the generation of Q, K, and V by constraining the attention computation to a fixed-size low-resolution query space and a multi-scale key-value space. On this basis, FQSA still adopts multi-head scaled dot-product attention for feature aggregation, while all attention operations are performed in the low-resolution space:


$$\begin{array}{c} \text{Attn}\left({𝐐}_{p},{𝐊}_{p},{𝐕}_{p}\right)=\text{Softmax}\left(\frac{{𝐐}_{p}{{𝐊}_{p}}^{⊤}}{\sqrt{d_{k}}}\right){𝐕}_{p} \end{array}$$ (1)

Where, ${𝐐}_{p}$, ${𝐊}_{p}$, and ${𝐕}_{p}$ are obtained from the low-resolution feature map through linear transformation. In FQSA, the sequence length of the query ${𝐐}_{p}$ is fixed at $N_{q}$, while the sequence lengths $N_{k}$ of keys ${𝐊}_{p}$ and ${𝐕}_{p}$ scale linearly with the input resolution. This design reduces the size of the attention matrix from N×N in standard self-attention to $N_{q}\times N_{k}$, thereby reducing memory consumption and improving computational efficiency.

The computational complexity of FQSA can be decomposed into several components. Specifically, the computational complexity of the adaptive pooling and upsampling operations is denoted as O(Nd), the complexity of the linear transformations as $O\left(md^{\text{2}}\right)$, and the complexity of the self-attention computation as $O\left(m^{\text{2}}d\right)$. Therefore, the overall computational complexity of FQSA can be expressed as $O(Nd+md^{\text{2}}+m^{\text{2}}d)$. Since m denotes the side length of the low-resolution query space and is fixed as a constant (e.g., m=16), independent of the input sequence length N, and d is much larger than m, the overall computational complexity can be simplified to $O(Nd+d^{\text{2}})$. Compared with the computational complexity $O(N^{\text{2}}d+Nd^{\text{2}})$ of standard self-attention, FQSA significantly reduces computational cost, enabling the model to efficiently handle high-resolution inputs.

In summary, by combining fixed-budget queries with multi-scale contextual keys and values, FQSA enables efficient attention computation within a compact representation space. This design not only preserves the ability to model global dependencies, but also effectively alleviates the excessive computational complexity of attention under high-resolution settings, thereby providing a practical solution for applying vision Transformers to dense prediction tasks.

### Large-Receptive-Field Enhancement (LREA)

**Fig 3:**
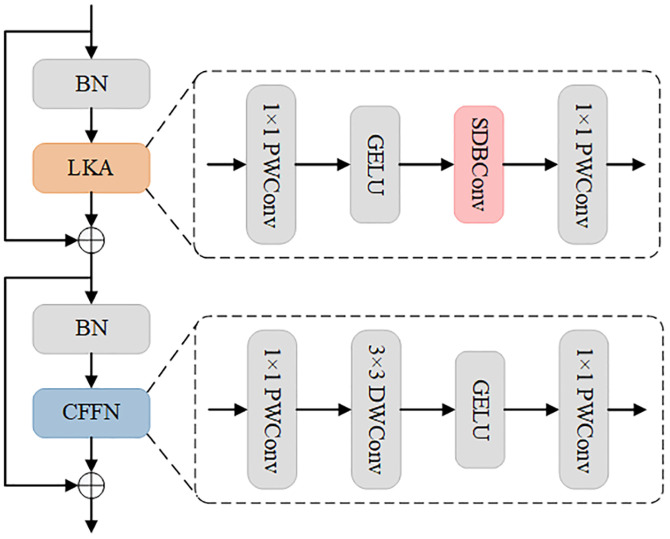
Architecture of LREA.

In UAV tiny-object detection, it is crucial to efficiently capture long-range contextual dependencies while preserving sensitivity to fine-grained features. Although existing architectures have made progress in long-range dependency modeling and receptive field expansion, several challenges remain. First, Vision Transformers [30] usually incur high computational cost, making them difficult to apply to efficient real-time processing. Second, efficient attention mechanisms such as SeaFormer [31] have reduced computational overhead to some extent, but they still face limitations in further performance optimization. In addition, large-kernel attention methods, such as SLaK [32] and LSKA [33], remain insufficient in terms of multi-scale feature fusion and dynamic receptive field adaptation. To address these issues, we propose a Large-Receptive-Field Enhancement (LREA) module, as illustrated in Fig 3. The design of LREA follows the mainstream attention-feedforward paradigm and mainly consists of two components: a Large Kernel Attention (LKA) mechanism and a Convolutional Feed-Forward Network (CFFN) [34]. Through the synergy of these two components, LREA expands the receptive field and captures global semantic information while effectively preserving fine details and boundary information, thereby improving both model accuracy and efficiency in UAV tiny-object detection.

LKA innovatively combines convolutional operations with the attention mechanism and expands the receptive field through the proposed Sparse Decomposed Broad Convolution (SDBConv) module. To further improve the adaptability of feature fusion, we design a Bidirectional Selective Aggregation (BSA) module, which enables the network to dynamically adjust the fusion of features at different scales according to the input content. The Convolutional Feed-Forward Network (CFFN) is responsible for further refining and integrating features, thereby ensuring a balanced output representation with sufficient information preservation. Specifically, CFFN adopts pointwise convolution (PWConv) to expand and compress the channel dimension, while a depthwise convolution (DWConv) is embedded in the intermediate stage to enhance spatial information interaction.

### Sparse Decomposed Broad Conv (SDBConv)

The SDBConv module is a core component of LREA. Through a sparse decomposition strategy, it decomposes a conventional large-kernel convolution into smaller convolutional units and two dilated convolution strips, thereby achieving an equivalent expansion of the receptive field.

**Fig 4:**
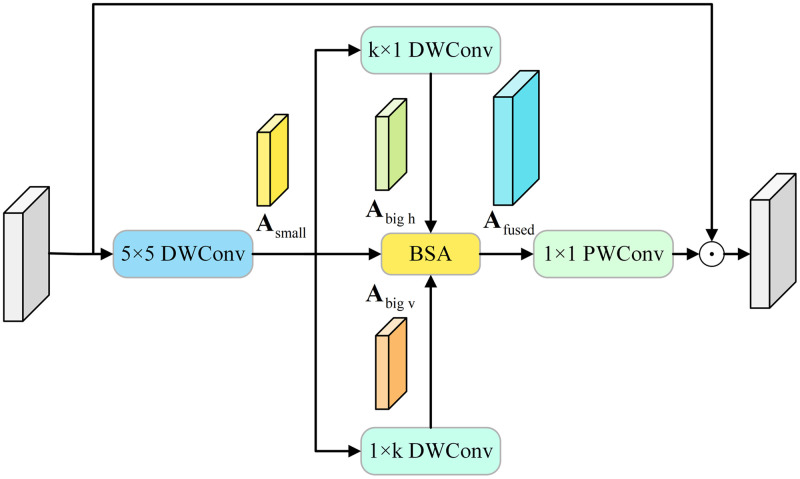
Architecture of SDBConv.

As illustrated in Fig 4, the SDBConv module first employs a 5 × 5 depthwise separable convolution to extract local features and generate a small-kernel attention feature map ${𝐀}_{\text{small}}$. The computational cost of this operation is substantially lower than that of directly using an extremely large convolution kernel. Subsequently, two strip-shaped dilated convolutions are applied in parallel to further process this feature map ${𝐀}_{\text{small}}$: one adopts a 1 × 𝑘 horizontal convolution, while the other uses a 𝑘 × 1 vertical convolution. This design enables the module to capture broader contextual information and expand the receptive field along the spatial dimension, thereby effectively enhancing long-range dependency modeling. The outputs of these two branches are denoted as the horizontally extended attention feature map ${𝐀}_{\text{big h}}$ and the vertically extended attention feature map ${𝐀}_{\text{big v}}$, respectively. These three complementary attention feature maps are then fed into the kernel selection mechanism for adaptive fusion, producing a fused attention feature map ${𝐀}_{\text{fused}}$. To further strengthen the representational capability of the fused feature, a pointwise convolution is finally applied to perform feature transformation and generate the modulation weight map. Finally, the generated weight map is multiplied element-wise with the original input to accomplish the final attention modulation. Overall, by virtue of this decomposition strategy, SDBConv significantly enlarges the receptive field while maintaining high computational efficiency, enabling effective long-range dependency modeling and providing efficient support for the large-kernel attention mechanism in LREA.

### Bidirectional Selective Aggregation (BSA)

**Fig 5:**
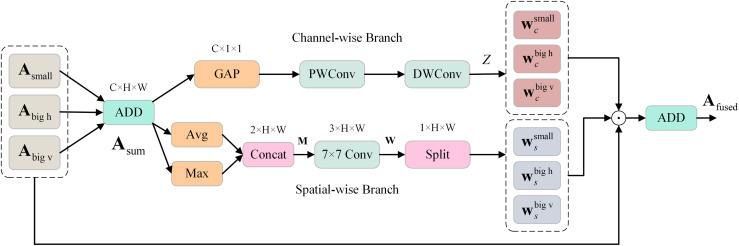
Architecture of BSA.

The three attention feature maps $\left\{{𝐀}_{\text{small}},{𝐀}_{\text{big h}}\text{,}{𝐀}_{\text{big v}}\right\}$ generated by SDBConv emphasize information at different scales and orientations, respectively. To effectively integrate these complementary features, we propose a Bidirectional Selective Aggregation (BSA) module. Within LREA, the BSA module dynamically adjusts feature weights along both the channel and spatial dimensions, thereby significantly enhancing the model’s feature fusion capability. The overall structure of BSA is illustrated in Fig 5.

Channel-weight branch: First, the three attention feature maps are summed to construct a shared attention feature ${𝐀}_{\text{sum}}\in {\mathbb{R}}^{B\times C\times H\times W}$:


$$\begin{array}{c} {𝐀}_{\text{sum}}={𝐀}_{\text{small}}+{𝐀}_{\text{big h}}+{𝐀}_{\text{big v}} \end{array}$$ (2)

Then, ${𝐀}_{\text{sum}}$ is processed by global average pooling, followed by lightweight pointwise convolution and depthwise convolution for feature mapping:


$$\begin{array}{c} Z=\text{DWConv}\left(\text{PWConv}\left(\text{GAP}\left(𝐀\right)\right)\right) \end{array}$$ (3)

Finally, three independent expansion mappings are applied to Z to generate three groups of channel weights ${𝐰}_{c}^{\text{small}}$, ${𝐰}_{c}^{\text{big h}}$ and ${𝐰}_{c}^{\text{big v}}$.

Spatial-weight branch: Average pooling and max pooling are applied to the shared attention feature ${𝐀}_{\text{sum}}$ along the channel dimension, and the resulting features are then concatenated:


$$\begin{array}{c} 𝐌=\text{Concat}\left(\text{AvgPool}\left({𝐀}_{\text{sum}}\right),\text{MaxPool}\left({𝐀}_{\text{sum}}\right)\right)\in {\mathbb{R}}^{B\times 2\times H\times W} \end{array}$$ (4)

Then, the feature map is fused and transformed through a 7 × 7 convolution to generate a spatial weight map:


$$\begin{array}{c} 𝐖={\text{Conv}}_{7\times 7}\left(𝐌\right)\in {\mathbb{R}}^{B\times 3\times H\times W}. \end{array}$$ (5)

𝐖 is divided into three groups, denoted as ${𝐰}_{s}^{\text{small}}$, ${𝐰}_{s}^{\text{big h}}$, and ${𝐰}_{s}^{\text{big v}}$. Finally, the channel weights, spatial weights, and the original attention maps are combined through element-wise multiplication and addition:


$$\begin{array}{c} {𝐀}_{\text{fused}}=\sum_{i\in \left\{\text{small},\text{big h},\text{big v}\right\}}{𝐀}_{i}⊙{𝐰}_{c}^{i}⊙{𝐰}_{s}^{i}\; \end{array}$$ (6)

The BSA module jointly models channel-wise and spatial-wise weights, enabling the network to dynamically adjust its receptive-field preference according to the input content. When target structures exhibit stronger horizontal continuity, ${𝐰}_{c}^{\text{big h}}$ and ${𝐰}_{s}^{\text{big h}}$ are enhanced; conversely, when richer vertical contextual information is required, ${𝐰}_{c}^{\text{big v}}$ and ${𝐰}_{s}^{\text{big v}}$ are enhanced. This mechanism allows for more expressive and adaptive multi-scale attention fusion.

LREA is particularly suitable for DETR-style detectors because the quality of encoder features directly affects query selection and subsequent bipartite matching. In UAV tiny-object detection, small targets are often weakly represented and easily confused with background textures. If the encoder features lack sufficient contextual discrimination, decoder queries may compete for ambiguous regions or be matched to redundant background responses. By expanding the receptive field through SDBConv and adaptively selecting contextual branches through BSA, LREA enhances object-relevant regions while preserving fine-grained spatial details. This improves the separability of tiny objects in the encoder memory and provides more reliable features for query initialization and matching in the decoder.

### Adaptive query modeling strategy

In the decoding stage, the D-FINE Transformer still adopts a fixed upper bound K on the number of queries for query selection and decoding. This design exhibits notable limitations in scenarios where the number of objects varies significantly across images. In sparse scenes, a fixed number of queries may introduce redundant predictions, leading to increased false positives and unnecessary computational overhead. In contrast, in dense scenes, the detection recall can be constrained by the upper bound K. To address these issues in a systematic manner, we introduce an adaptive query modeling strategy within the AQF-Net framework. Inspired by the concept of Dynamic Query Selection [26], this strategy leverages predicted object count information to adaptively determine the number of queries required by the decoder, thereby enabling dynamic alignment between query allocation and scene complexity.

**Fig 6:**
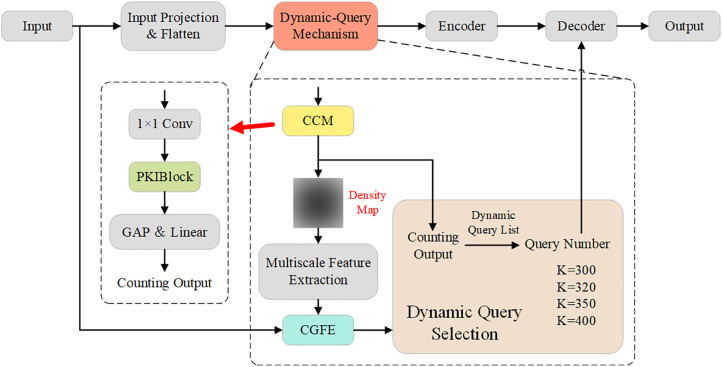
Architecture of adaptive query modeling strategy.

The overall architecture of the adaptive query modeling strategy is illustrated in Fig 6, which consists of three key components: the Categorical Counting Module (CCM), the Counting-Guided Feature Enhancement (CGFE) module, and the Dynamic Query Selection module. In particular, the PKIBlock from PKINet [35] is incorporated into the CCM to enhance multi-scale feature extraction and improve the robustness of object counting. The processing pipeline is as follows. First, the input features are processed through Projection and Flatten operations and then fed into the dynamic query mechanism. The CCM predicts the number of objects in the image and uses this information to estimate the number of queries required by the decoder. The predicted count is subsequently mapped to a specific query number through the dynamic query mapping process, which determines the actual number of queries used during decoding. Meanwhile, the CGFE module performs multi-scale weighted fusion on the density map generated by the CCM to further enhance feature representation. Finally, by dynamically adjusting the number of queries, the model adaptively allocates computational resources across different scenarios, thereby improving both detection performance and efficiency.

### Categorical counting module (CCM)

Within the adaptive query modeling strategy, the Categorical Counting Module (CCM) aims to predict the number of object instances in the input image and categorize them into predefined density levels, which are then used to guide dynamic query allocation. To enhance the representational capability of counting features while maintaining computational efficiency, a lightweight design is adopted for CCM by introducing efficient channel encoding and a multi-kernel convolution module, namely PKIBlock.

**Fig 7:**
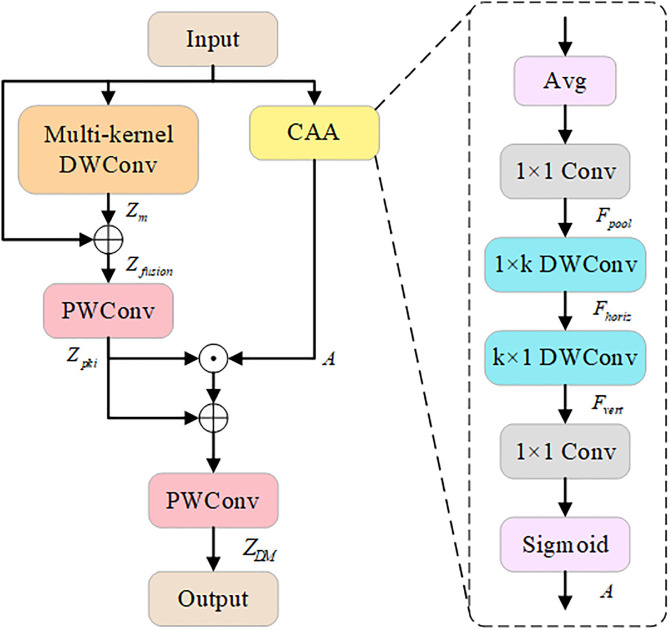
Architecture of PKIBlock.

As illustrated in Fig 7, PKIBlock consists of two key submodules: a multi-scale depthwise convolution aggregation module and a Context Anchor Attention (CAA) module. The former captures local contextual information at multiple scales, while the latter incorporates global contextual cues to further enhance feature representation, thereby improving the accuracy and robustness of object counting.

The detailed computation process is as follows. Let the input feature be denoted as $X\in R^{C_{in}\times H\times W}$. The multi-scale depthwise convolution aggregation module adopts an Inception-style multi-branch structure, where depthwise separable convolutions with different kernel sizes are applied in parallel to capture local contextual information at multiple scales. Specifically, M  kernel sizes are selected, denoted as $\{k_{m}{\}}_{m=1}^{M}$. For each kernel size $k_{m}$, the following operation is performed:


$$\begin{array}{c} Z_{m}={\text{DWConv}}_{k_{m}\times k_{m}}\left(Z_{\text{0}}\right)\in {\mathbb{R}}^{C_{in}\times H\times W},\text{\textbackslash{}hspace\{1em}\}m=1,\ldots ,M \end{array}$$ (7)

Since each convolution operates independently on each input channel, this design significantly reduces the number of parameters. The outputs from all multi-scale branches are then fused with the original input feature $Z_{\text{0}}$ via element-wise addition. Subsequently, a 1 × 1 pointwise convolution is applied to the fused feature to enable inter-channel interaction and dimensional compression:


$$\begin{array}{c} Z_{pki}={\text{PWConv}}_{1\times 1}(Z_{\text{0}}+\sum_{m=1}^{M}Z_{m})\in {\mathbb{R}}^{C_{in}\times H\times W} \end{array}$$ (8)

To capture long-range global dependencies, PKIBlock introduces a Context Anchor Attention (CAA) branch, which generates channel attention weights through global information aggregation and lightweight mapping. First, the input feature $Z_{\text{0}}$ is processed by global average pooling, followed by channel transformation to obtain a compact contextual representation. Subsequently, two depthwise separable convolutions are applied along the horizontal and vertical directions, respectively, to spatially propagate the context vector, enabling effective dissemination of global information.


$$\begin{array}{c} \begin{array}{c} F_{horiz}\;={\text{DWConv}}_{1\times k_{h}}\;\left({\text{Conv}}_{1\times 1}\left(\text{AvgPool}\left(Z_{\text{0}}\;\right)\right)\;\right)\\ F_{vert}\;={\text{DWConv}}_{k_{v}\;\times 1}\;\left(F_{horiz}\;\right) \end{array} \end{array}$$ (9)

Here, $k_{h}$ and $k_{v}$ denote the kernel sizes along the horizontal and vertical directions, respectively. This cascaded strip convolution design enables the module to capture spatial contextual information within a large receptive field while maintaining low parameter and computational overhead. Finally, the CAA branch produces a channel attention weight map $A\in {\mathbb{R}}^{C_{in}\times H\times W}$.


$$\begin{array}{c} A=\sigma \left({\text{Conv}}_{1\times 1}\;\left(F_{vert}\;\right)\right) \end{array}$$ (10)

After obtaining the multi-scale convolution feature $Z_{pki}$ and the channel attention weight A, PKIBlock integrates them through a gated residual fusion mechanism, resulting in the final density map $Z_{DM}$.


$$\begin{array}{c} Z_{DM}={\text{PWConv}}_{1\times 1}\left(A⊙Z_{pki}\;⊕Z_{pki}\right)\; \end{array}$$ (11)

After being processed by the PKIBlock, the resulting density map $Z_{DM}$ serves two purposes. On the one hand, it is fed into a global average pooling layer followed by a fully connected layer to produce the counting output, which is used to determine the number of queries in the subsequent dynamic query selection. On the other hand, the density map is treated as an intermediate feature and forwarded to the CGFE module for further feature enhancement.

### Counting-guided feature enhancement module (CGFE)

**Fig 8:**
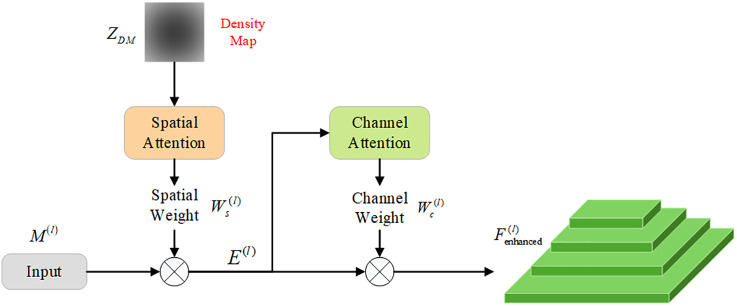
Architecture of CGFE.

The CGFE module is an important component of the adaptive query modeling strategy and leverages the spatial prior information embedded in the density map generated by the CCM to enhance feature responses in object-relevant regions of the encoder features. It consists of two components, namely spatial attention and channel attention. Guided by the density map, CGFE employs a dual-attention mechanism to refine feature representations. The overall architecture of CGFE is illustrated in Fig 8:

**Spatial attention**: First, the CGFE module employs a spatial attention mechanism to capture important spatial locations in the input feature maps. Let $M^{\left(l\right)}\in {\mathbb{R}}^{C\times H_{l}\times W_{l}}$ denote the multi-scale feature maps output by the neck, where l=1,...,L represents the number of feature levels. The density map $Z_{DM}$ is resized to match the spatial resolution of each feature level via a 1 × 1 convolution followed by interpolation, resulting in aligned density features $Z_{DM}^{\left(l\right)}\in {\mathbb{R}}^{C\times H_{l}\times W_{l}}$. For each level l, the spatial attention weights are computed as follows:


$$\begin{array}{c} W_{s}^{\left(l\right)}=\sigma \left({\text{Conv}}_{7\times 7}\left(\text{Concat}\left(\text{AvgPool}\left(Z_{DM}^{\left(l\right)}\right),\text{MaxPool}\left(Z_{DM}^{\left(l\right)}\right)\right)\right)\right)\in {\mathbb{R}}^{1\times H_{l}\times W_{l}} \end{array}$$ (12)

The spatial attention weights are then applied to the corresponding feature maps via element-wise multiplication, producing spatially enhanced features:


$$\begin{array}{c} E^{\left(l\right)}=W_{s}^{\left(l\right)}⊗M^{\left(l\right)}\in {\mathbb{R}}^{C\times H_{l}\times W_{l}} \end{array}$$ (13)

**Channel attention**: After spatial attention refinement, CGFE further performs channel attention on the spatially enhanced feature $E^{\left(l\right)}$:


$$\begin{array}{c} W_{c}^{\left(l\right)}=\sigma \left(\text{MLP}\left(\text{AvgPool}\left(E^{\left(l\right)}\right)\right)+\text{MLP}\left(\text{MaxPool}\left(E^{\left(l\right)}\right)\right)\right)\in {\mathbb{R}}^{C\times 1\times 1} \end{array}$$ (14)

The resulting channel attention weights $W_{c}^{\left(l\right)}$ are then multiplied element-wise with the spatially enhanced features $E^{\left(l\right)}$ to obtain the final enhanced features at each level:


$$\begin{array}{c} F_{\text{enhanced}}^{\left(l\right)}=W_{c}^{\left(l\right)}⊗E^{\left(l\right)} \end{array}$$ (15)

Finally, the enhanced features $F_{\text{enhanced}}^{\left(l\right)}$ are fed into the Dynamic Query Selection module, where they are used to compute objectness scores for query selection and to generate the content and positional embeddings of the initial queries.

### Dynamic query selection

As the final component of the adaptive query modeling strategy, the Dynamic Query Selection module adaptively determines the number of queries and their initial positions for each image based on the predicted object count and the enhanced features. For query content and positional initialization, the initial queries are derived from the enhanced features $F_{\text{enhanced}}$ produced by the CGFE module. Specifically, the multi-scale enhanced features $F_{\text{enhanced}}$ are flattened into a two-dimensional feature sequence $F_{flat}\in {\mathbb{R}}^{C\times \left(H_{\text{1}}W_{\text{1}}+...+H_{l}W_{l}\right)}$, and a feed-forward network is employed to compute objectness scores for each spatial location. The Top-K features with the highest scores are then selected to form a candidate feature set $F_{\text{select}}$:


$$\begin{array}{c} F_{\text{select}}=\text{topk}\left({\text{FFN}}_{\text{score}}\left(F_{\text{enhanced}}\right)\;,K\right) \end{array}$$ (16)

The candidate feature set $F_{\text{select}}$ contains rich semantic information, which is further transformed via a linear projection to generate the content embeddings of the queries:


$$\begin{array}{c} Q_{\text{content}}={\text{Linear}}_{\text{content}}\left(F_{\text{select}}\right) \end{array}$$ (17)

Each selected feature point corresponds to a spatial location in the original feature map. Its normalized coordinates $\left(x_{i},y_{i}\right)$, together with predefined anchor box sizes $\left(w_{i}\;,h_{i}\;\right)$, are used as the initial positional priors to construct reference anchors $A_{\text{selected}}={\left\{(x_{i},y_{i},w_{i},h_{i})\right\}}_{i=1}^{K}$. These anchors are further refined by predicting offset adjustments through a feed-forward network (FFN), resulting in the final positional priors:


$$\begin{array}{c} Q_{\text{position}}=A_{\text{selected}}+{\text{FFN}}_{\text{bbox}}\left(F_{\text{select}}\right) \end{array}$$ (18)

The resulting dynamic queries are then fed into the decoder for iterative refinement, and the final detection results are produced by the prediction head.

### Experiments

#### Dataset description.

The proposed method is evaluated on three datasets, including CODrone, VisDrone2019, and a self-constructed PV-DV dataset.

**Fig 9:**
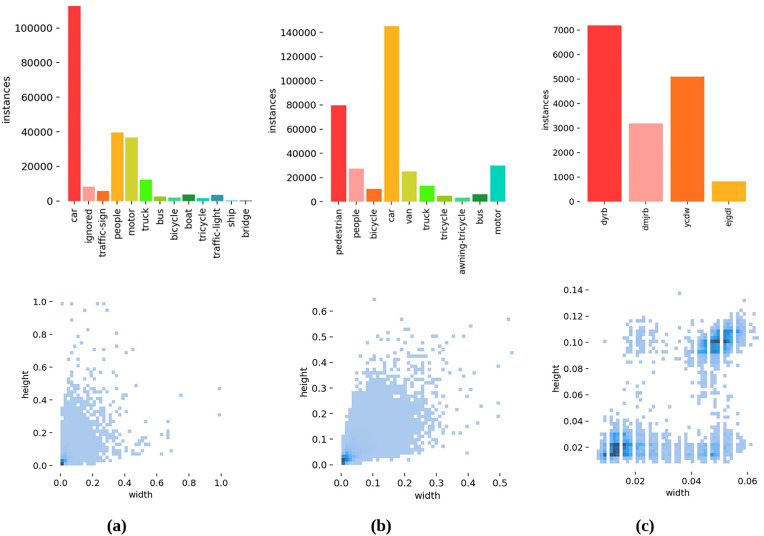
Datasets label distribution map from the (a) CODrone, (b) VisDrone2019, and (c) PV-DV datasets.

CODrone [36] is released by Xiamen University and is designed for oriented object detection in UAV aerial imagery under real-world flight conditions. It contains 10,004 images with annotations covering 12 categories of typical urban and traffic-related objects, including vehicles, traffic facilities, pedestrians, light transportation tools, and vessels. The dataset is split into training, validation, and test sets with a ratio of 5:2:3. Sample images and annotation statistics are illustrated in Fig 9(a).

VisDrone2019 [37], constructed by the AISKYEYE team from Tianjin University, is a large-scale UAV-based benchmark dataset. All images are collected from 14 cities in China and cover diverse scenarios such as traffic intersections, urban environments, and crowded areas. The dataset contains 8,629 images, which are divided into 6,471 training images, 548 validation images, and 1,610 test images. It includes 10 object categories, such as pedestrians, cars, bicycles, tricycles, and buses. In addition to object locations and categories, annotations also include attributes such as occlusion levels and lighting conditions. Sample images and annotation statistics are shown in Fig 9(b).

PV-DV is an infrared photovoltaic defect dataset constructed by our team to support UAV-based inspection and defect detection in large-scale photovoltaic power plants. The dataset initially contains 699 infrared images and is expanded to 1398 images through data augmentation to improve sample diversity and model robustness. It includes four typical defect types: single hot spots, large-area hot spots, abnormal low-temperature regions, and diode short circuits. Specifically, there are 15716 instances of single hot spots, 1336 large-area hot spots, 11804 abnormal low-temperature defects, and 2888 diode short circuits. These annotations comprehensively reflect the diversity and complexity of real-world photovoltaic defect distributions. Based on the width–height distribution of bounding boxes, the dataset is well suited for tiny-object detection. It is divided into training, validation, and test sets with a ratio of 7:2:1, ensuring a balanced distribution of defect categories across subsets. Sample images and annotation statistics are shown in Fig 9(c).

**Fig 10:**
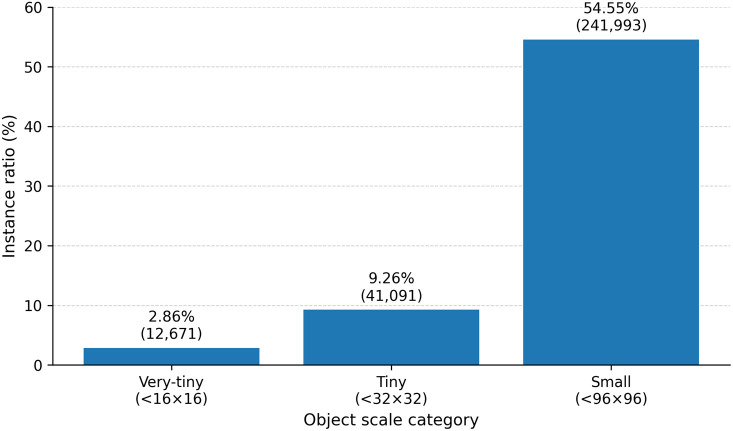
Object scale distribution of the CODrone dataset. The categories are defined according to COCO-style area thresholds and AI-TOD-inspired relative-area analysis.

To further clarify the object scale distribution of the CODrone dataset, we analyze the relative area of annotated instances following the COCO and AI-TOD evaluation protocols. As shown in Fig 10, 2.86% of instances fall into the very-tiny category, 9.26% fall into the tiny category, and 54.55% belong to the small-object category.

These results indicate that CODrone is highly dominated by small-scale and tiny-scale objects, which are consistent with real-world UAV aerial imaging scenarios. This distribution further validates the suitability of CODrone for evaluating UAV tiny-object detection methods.

### Implementation details

All experiments are conducted on a hardware platform equipped with an Intel Xeon Platinum 8470Q CPU (2.10 GHz) and an NVIDIA RTX 4090 GPU with 24 GB memory. The software environment includes Ubuntu 22.04, Python 3.12, PyTorch 2.3, and CUDA 12.1. During training, a flat-cosine learning rate schedule is adopted, where the first 500 iterations are used for linear warm-up. After the 72nd epoch, strong data augmentation strategies are gradually disabled, and only basic augmentations are retained to facilitate stable convergence. Unless otherwise specified, all hyperparameters follow the default settings of the official implementation. Detailed experimental configurations are provided in Table 1.

**Table 1:** Experimental settings.

parameter	Set value
Epochs	128
Batch size	4
Image size	640 × 640
Initial learning rate	0.0002
Optimizer	AdamW
Momentum β_1_, β_2_	[0.9,0.999]
Weight decay	0.0001

### Ablation experiments

To validate the effectiveness of the proposed components, ablation experiments are conducted on the CODrone dataset, and the results are presented in Table 2. Using D-FINE-M as the baseline, we progressively incorporate different components and analyze the resulting changes in detection performance and computational complexity. Unless otherwise specified, all FPS and latency results in the following tables are measured under the same PyTorch FP16 inference setting with batch size 4 and an input resolution of 640 × 640.

**Table 2:** Ablation experiments on the CODrone dataset. AQM denotes the adaptive query modeling strategy.

Methods	FQSA	LREA	AQM	AP	AP_50_	AP_m_	Params	GFLOPs	FPS
Baseline				16.4	30.9	16.0	19.19	56.3	118.5
	✓			17.1	33.0	17.2	18.67	55.8	133.3
		✓		17.2	32.7	17.5	21.55	68.8	98.7
			✓	17.0	32.7	17.1	20.86	64.8	104.0
	✓	✓		17.2	33.5	17.0	21.04	68.3	104.2
	✓		✓	17.4	33.7	17.5	20.34	64.3	120.4
		✓	✓	17.3	33.3	17.3	23.22	77.2	102.3
	✓	✓	✓	17.8	34.4	17.6	22.70	76.7	85.7

Specifically, when only the Fixed-Query Self-Attention (FQSA) mechanism is introduced, the AP improves from 16.4 to 17.1, while the number of parameters and computational cost decrease to 18.67M and 55.8 GFLOPs, respectively. Meanwhile, the FPS increases from 118.5 to 133.3, indicating that FQSA improves inference efficiency while still providing stable performance gains. After incorporating the LREA module, the AP further increases to 17.2, with AP_m_ reaching 17.5. This suggests that expanding the receptive field and enhancing multi-scale feature fusion can effectively improve the representation capability for tiny objects, although the computational cost correspondingly rises to 68.8 GFLOPs and the FPS decreases to 98.7. When only the adaptive query modeling strategy is introduced, the AP reaches 17.0, validating the effectiveness of dynamic query allocation and enhanced feature interaction in improving target matching quality, with an FPS of 104.0. More importantly, combining multiple components further exploits their complementary benefits. When FQSA, LREA, and the adaptive query modeling strategy are jointly integrated, the model achieves the best performance, reaching 17.8 AP, 34.4 AP_50_, and 17.6 AP_m_, while maintaining 85.7 FPS. Compared with the baseline, these results demonstrate consistent improvements in both overall detection accuracy and tiny-object detection performance. Although the multi-scale feature enhancement and dynamic query mechanism introduce additional computational overhead, the model still maintains practical inference speed, reflecting a favorable trade-off between detection accuracy and computational efficiency. It should be noted that, according to the COCO evaluation protocol, AP_s_ is commonly used to evaluate tiny-object detection performance. However, on the CODrone dataset, the AP_s_ values are observed to be extremely low and highly unstable, making it difficult to effectively distinguish the performance differences among methods. Therefore, AP_m_ is adopted in this work as a more stable alternative metric.

To further validate the generalizability of the above findings across different aerial scenarios, additional ablation experiments are conducted on the VisDrone training set to examine the stability and effectiveness of AQF-Net under different data distributions. The experimental results are summarized in Table 3.

**Table 3:** Ablation experiments on the VisDrone dataset. AQM denotes the adaptive query modeling strategy.

Methods	FQSA	LREA	AQM	AP	AP_50_	AP_s_	Params	GFLOPs	FPS
Baseline				28.7	48.0	17.3	19.19	56.3	120.5
	✓			29.7	49.8	17.9	18.67	55.8	127.7
	✓	✓		30.7	50.9	19.1	21.04	68.3	101.8
	✓	✓	✓	33.1	54.6	21.2	22.70	76.7	78.3

According to Table 3, with the progressive introduction of FQSA, LREA, and the adaptive query modeling strategy, AP steadily increases from the baseline value of 28.7 to 33.1, while AP_50_ and AP_s_ reach 54.6 and 21.2, respectively. In terms of inference speed, FQSA increases the FPS from 120.5 to 127.7, further confirming its efficiency advantage. After introducing LREA and the full adaptive query modeling strategy, the FPS decreases to 101.8 and 78.3, respectively, due to the additional feature enhancement and query allocation operations. Nevertheless, the full AQF-Net still maintains real-time inference capability while achieving substantial accuracy gains. These results indicate that each component effectively improves both overall detection performance and tiny-object detection capability, while maintaining a favorable balance between accuracy and efficiency. The ablation results further confirm the effectiveness of the proposed components across different scenarios and demonstrate their complementary roles within the AQF-Net framework.

To evaluate the statistical reliability of the performance improvement, we conduct three independent runs with different random seeds on the VisDrone2019 validation set. The D-FINE baseline and AQF-Net are trained and evaluated under the same experimental settings. We report the mean and standard deviation of AP, AP_50_, and AP_75_, and perform a two-sided paired t-test between the two methods.

As shown in Table 4, AQF-Net consistently outperforms the D-FINE baseline across all three runs. The improvements in AP, AP_50_, and AP_75_ are 5.27%, 7.20%, and 6.13%, respectively, with all p-values below 0.01. These results indicate that the performance gains of AQF-Net are statistically reliable and not merely caused by random seed variation.

**Table 4:** Statistical significance analysis on the VisDrone2019 validation set over three independent runs.

Methods	AP	AP_50_	AP_75_
D-FINE baseline	28.53 ± 0.15	47.70 ± 0.26	28.93 ± 0.15
AQF-Net	33.80 ± 0.62	54.90 ± 0.36	35.07 ± 0.68
Improvement	+5.27	+7.20	+6.13
p-value	0.0068	0.0023	0.0061

### Comparative experiments

The comparative results on the VisDrone validation set are reported in Table 5. Overall, conventional general-purpose detectors, such as YOLO and DETR, suffer from evident performance bottlenecks in this challenging aerial scenario. Even when the model scale is enlarged or deformable attention mechanisms are introduced, the improvement in detection accuracy remains limited and is often accompanied by a substantial increase in model parameters and computational cost. In contrast, methods specifically designed for UAV tiny-object detection demonstrate more competitive performance. Approaches such as QueryDet, SINextNet, SOD-YOLO, UAV-DETR, and SO-DETR improve detection accuracy by introducing mechanisms including dense sampling, hierarchical feature reweighting, and query-level modeling. However, some of these methods, such as UAV-DETR, SO-DETR, and FBRT-YOLO-X, rely on highly complex Transformer decoding structures or multi-branch feature interaction designs, resulting in a significant increase in parameter size and computational complexity. This imposes considerable deployment pressure in practical UAV platforms and real-time application scenarios. Overall, existing methods still struggle to achieve an ideal balance between detection accuracy and computational efficiency. This further highlights the necessity and research value of structured modeling and query optimization for aerial tiny-object detection under controllable computational overhead. The proposed AQF-Net-M achieves the best performance on the VisDrone validation set, reaching 33.4 AP, 55.0 AP_50_, and 33.9 AP_75_, while maintaining a controllable model size of 22.70M parameters and a computational cost of 76.7 GFLOPs. These results significantly outperform conventional general-purpose detectors as well as most UAV-specific methods, demonstrating the effectiveness and superiority of the proposed adaptive query modeling and feature interaction mechanisms in complex aerial tiny-object detection scenarios.

**Table 5:** Comparison of methods on the VisDrone validation set.

Methods	AP	AP_50_	AP_75_	Params	GFLOPs
YOLOv8-S [38]	22.3	37.6	—	11.16	28.8
YOLOv8-M [38]	24.6	40.7	—	25.90	79.3
YOLOv9-S [39]	22.7	38.3	—	7.20	26.7
YOLOv9-M [39]	25.2	42.0	—	20.10	76.8
YOLOv10-S [40]	22.2	37.4	—	7.22	21.4
YOLOv10-M [40]	24.5	40.5	—	15.32	58.9
YOLOv11-S [41]	23.0	38.7	—	9.42	21.3
YOLOv11-M [41]	25.9	43.1	—	20.04	67.7
RTDETR-R18 [12]	26.5	44.0	26.7	20.18	58.6
Deformable DETR [42]	27.1	42.2	—	40.02	173.0
QueryDet [43]	28.3	48.1	28.7	—	—
SINextNet [44]	29.9	48.1	—	49.50	51.8
FBRT-YOLO-X [23]	30.1	48.4	31.7	22.82	185.8
YOLC [45]	28.9	51.4	28.3	—	—
CEASC [46]	28.7	50.7	28.4	21.52	150.1
SOD-YOLO [47]	30.0	50.7	—	30.35	83.5
UAV-DETR [48]	31.5	51.1	—	42.48	170.0
SO-DETR [49]	31.5	51.5	—	44.42	161.4
D-FINE-S [14]	27.4	46.1	—	10.18	24.8
D-FINE-M [14]	28.8	48.1	29.0	19.19	56.3
DEIM-D-FINE-S [50]	27.1	45.7	—	10.18	24.8
DEIM-D-FINE-M [50]	29.9	48.5	—	19.19	56.3
AQF-Net-S	31.4	52.1	31.8	14.98	55.0
AQF-Net-M	33.4	55.0	33.9	22.70	76.7

To evaluate the performance of different detection methods on photovoltaic array defect detection, comparative experiments are conducted on the PV-DV dataset, and the results are presented in Table 6. The results show that AQF-Net-M achieves the best performance in terms of AP, AP_50_, and AP_75_, reaching 58.7%, 85.5%, and 62.3%, respectively, significantly outperforming the other competing methods. In terms of inference efficiency, AQF-Net-M achieves 70.2 FPS with a latency of 14.24 ms, indicating that the improved accuracy is obtained at the cost of additional computational overhead. As a lightweight version of AQF-Net-M, AQF-Net-S is designed for resource-constrained scenarios by reducing the number of internal channels in the model. Despite its lower complexity, it still demonstrates strong detection performance, achieving 57.5% AP, 84.5% AP_50_, and 61.6% AP_75_, while improving the inference speed to 109.3 FPS with a latency of 9.15 ms. Overall, the AQF-Net series outperforms other mainstream detection methods on the PV-DV dataset, further validating the effectiveness and application potential of the proposed method for photovoltaic array defect detection.

**Table 6:** Comparison of methods on the PV-DV dataset.

Methods	AP	AP_50_	AP_75_	Params	GFLOPs	FPS	Latency (ms)
YOLOv8-S	54.1	78.0	56.1	11.16	28.8	167.5	5.97
YOLOv8-M	56.5	79.9	59.9	25.90	79.3	135.6	7.37
YOLOv9-S	51.2	75.9	55.3	7.20	26.7	169.2	5.91
YOLOv9-M	54.3	81.2	57.0	20.10	76.8	143.2	6.98
YOLOv10-S	50.3	75.3	51.8	7.22	21.4	190.8	5.24
YOLOV10-M	52.8	76.7	55.5	15.32	58.9	156.7	6.38
YOLOv11-S	52.6	80.0	54.2	9.42	21.3	200.4	4.99
YOLOv11-M	55.2	80.7	57.7	20.04	67.7	156.4	6.39
RTDETR-R18	56.3	83.5	60.2	20.18	58.6	104.2	9.59
D-FINE-S	55.6	82.9	57.3	10.18	24.8	150.5	6.64
D-FINE-M	56.9	83.8	60.5	19.19	56.3	124.0	8.06
AQF-Net-S	57.5	84.5	61.6	14.98	55.0	109.3	9.15
AQF-Net-M	58.7	85.5	62.3	22.70	76.7	70.2	14.24

### Roles of key innovations

#### Effect of adaptive query allocation.

To investigate the impact of different configurations of the dynamic query mechanism within the adaptive query modeling strategy, systematic ablation experiments are conducted on the CODrone dataset based on the D-FINE-M architecture. While keeping the core idea of dynamic query allocation and the overall decoding pipeline unchanged, these experiments only modify the Categorical Counting Module through lightweight redesign or structural enhancement. The experimental results are summarized in Table 7. For clarity, the final configuration with the lightweight counting module and PKIBlock is referred to as the proposed adaptive query modeling strategy in AQF-Net, whereas the other two configurations are intermediate variants used only for ablation analysis.

**Table 7:** Ablation experiments on the dynamic query mechanism.

Methods	AP	AP_50_	AP_75_	Params	GFLOPs
Baseline	16.4	30.9	15.7	19.19	56.3
Baseline + Original Dynamic Query Mechanism	16.5	31.6	15.2	24.05	105.7
Baseline + Lightweight Counting Module	16.4	31.8	15.2	21.63	74.7
Baseline + Proposed Adaptive Query Modeling Strategy	17.0	32.7	15.9	21.55	68.8

As shown in Table 7, after introducing the dynamic query mechanism into the D-FINE-M baseline, the overall detection performance improves steadily, but the number of parameters and computational cost also increase significantly. Based on this observation, we further perform structural ablation analysis on the Categorical Counting Module. The results show that the lightweight design effectively reduces computational overhead while maintaining detection accuracy, indicating that the original counting module in the dynamic query mechanism contains a certain degree of structural redundancy. Furthermore, after incorporating PKIBlock, the model achieves the best performance in terms of AP, AP_50_, and AP_75_, while introducing no significant additional computational cost compared with the baseline. These results demonstrate that proper optimization of the internal structure of the dynamic query mechanism helps achieve a better balance between detection accuracy and computational efficiency.

To verify whether the improvement of AQM comes from density-aware query allocation rather than merely varying the number of decoder queries, we conducted controlled ablation experiments on the VisDrone2019 validation set. In particular, we introduce a random query masking baseline. In this setting, the number of decoder queries is randomly sampled from the same candidate query list, i.e., [300, 330, 360, 400], while the CCM modules are retained. Therefore, random query masking preserves the effect of using dynamic query numbers but removes the density-aware allocation rule.

As shown in Table 8, compared with the D-FINE baseline using a fixed number of 300 queries, introducing CCM while keeping the query number unchanged improves AP from 28.7% to 31.5%, AP_50_ from 48.0% to 52.1%, and AP_75_ from 28.4% to 31.8%. This indicates that the counting-guided feature enhancement provides effective density-aware priors for improving feature representation. However, when the number of queries is randomly selected from [300, 330, 360, 400], the model achieves only 30.2% AP, which is lower than the fixed-query CCM variant and clearly inferior to the full AQM. This suggests that varying the number of decoder queries alone does not consistently improve detection performance and may even introduce mismatches between scene density and query allocation. In contrast, the full AQM achieves the best performance, with 33.1% AP, 54.6% AP_50_, and 33.7% AP_75_. These results demonstrate that the improvement of AQM is not merely caused by changing or reducing the number of queries, but mainly benefits from the density-aware query allocation guided by the CCM, which adaptively aligns the query budget with scene-level object density and alleviates redundant background predictions and query competition in UAV tiny-object detection.

**Table 8:** Ablation study of adaptive query allocation on the VisDrone2019 validation set.

Methods	Number of queries	AP	AP_50_	AP_75_
D-FINE baseline	300	28.7	48.0	28.4
Fixed queries + CCM	300	31.5	52.1	31.8
Random query masking	[300, 330, 360, 400]	30.2	49.6	30.9
AQM	[300, 330, 360, 400]	33.1	54.6	33.7

To further evaluate the reliability of the Categorical Counting Module (CCM), we analyze its density prediction and query allocation behavior on the VisDrone2019 validation set. According to the number of annotated instances in each image, the ground-truth density levels are divided into four categories: sparse (≤10 objects), moderate (11–60 objects), dense (61–150 objects), and very dense (>150 objects). These four density levels are mapped to 300, 330, 360, and 400 decoder queries, respectively.

**Fig 11:**
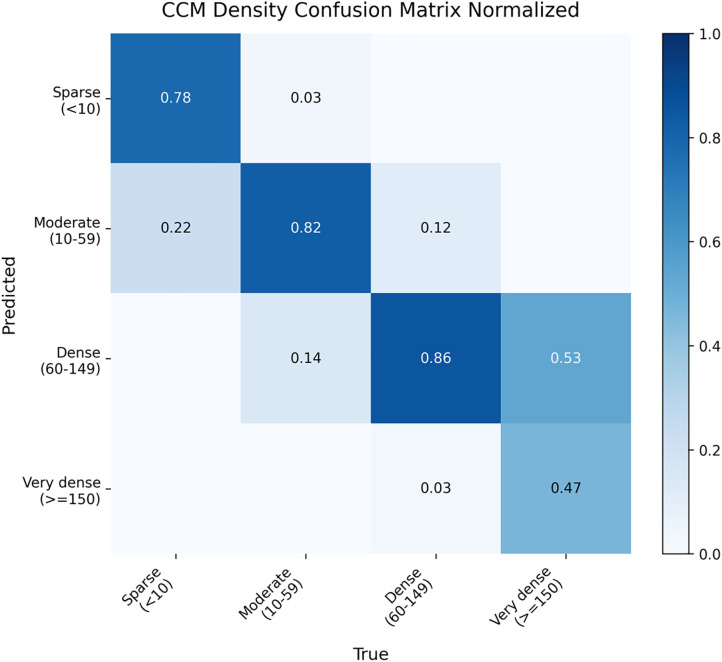
Normalized confusion matrix of CCM density prediction on the VisDrone2019 validation set.

As shown in Table 9, CCM achieves 81.75% density classification accuracy and 100.00% adjacent accuracy. The under-allocation and over-allocation rates are 10.04% and 8.21%, respectively, while the query MAE and RMSE are 5.91 and 13.96. The confusion matrix in Fig 11 further shows that all misclassified samples fall into adjacent density categories, and no severe cross-level prediction error occurs. These results indicate that CCM provides stable density-aware guidance for dynamic query allocation, even when the predicted density level is not exactly correct.

**Table 9:** Reliability analysis of CCM on the VisDrone2019 validation set.

Metric	Value
Number of validation images	548
Density classification accuracy (%)	81.75
Adjacent accuracy (%)	100.00
Under-allocation rate (%)	10.04
Over-allocation rate (%)	8.21
Query MAE	5.91
Query RMSE	13.96
Mean prediction confidence (%)	83.80

### Effect of query-space resolution in FQSA

To investigate whether the fixed low-resolution query space in FQSA affects the localization accuracy of tiny objects, we conduct a sensitivity analysis by varying the query resolution while keeping all other settings unchanged. Specifically, we evaluate three query resolutions (8, 16, and 24) and compare them with the original D-FINE baseline.

As shown in Table 10, introducing FQSA with an excessively small query resolution (8) slightly decreases detection performance, particularly under the stricter AP_75_ metric, indicating that excessive query compression may weaken fine-grained localization. Increasing the query resolution to 16 significantly improves AP, AP_50_, and AP_75_, achieving the best overall performance with 17.2% AP and 16.0% AP_75_. Although a larger query resolution (24) further improves AP_75_ to 16.3%, the overall AP drops slightly while the computational cost increases from 55.8 GFLOPs to 57.1 GFLOPs. These results demonstrate that FQSA effectively balances efficiency and localization accuracy by performing attention computation in a fixed low-resolution query space. The default setting of query resolution 16 provides the most favorable trade-off between detection performance and computational complexity, and is therefore adopted in the final model.

**Table 10:** Effect of query resolution in FQSA on the VisDrone2019 validation set.

Methods	Query resolution	AP	AP_50_	AP_75_	GFLOPs
D-FINE baseline	–	16.4	30.9	15.3	56.3
D-FINE+FQSA	8	16.1	30.5	14.6	54.7
D-FINE+FQSA	16	17.2	32.7	16.0	55.8
D-FINE+FQSA	24	16.8	31.9	16.3	57.1

### Effect of LREA

To further verify the effectiveness and distinctiveness of the proposed LREA module at the neck feature enhancement stage, we conduct comparative experiments on the CODrone dataset. The LREA module at the position shown in Fig 1 is replaced with several representative feature enhancement and large-kernel context modules, including RGCSPELAN, CSSC, CFBlock, FCM, FMB, InceptionNeXt Block, CNCM, LKA, LSKA, and StripConv. In addition, SDBConv is evaluated separately to examine the contribution of the proposed sparse decomposed broad convolution, while the full LREA is used to evaluate the effect of combining SDBConv with bidirectional selective aggregation. All modules are inserted at the same position and trained under identical settings. The experimental results are summarized in Table 11.

**Table 11:** Comparison with other feature enhancement modules.

Methods	AP	AP_50_	AP_75_	Params	GFLOPs
Baseline	16.4	30.9	15.7	19.19	56.3
Baseline with RGCSPELAN	15.5	30.2	14.1	14.68	33.9
Baseline with CSSC	16.3	31.7	14.9	25.61	58.4
Baseline with CFBlock	16.9	32.2	15.8	19.58	62.9
Baseline with FCM	16.5	31.5	15.6	16.20	41.7
Baseline with FMB	16.4	31.6	15.4	18.24	50.2
Baseline with InceptionNeXt Block	15.8	30.2	14.9	16.84	43.1
Baseline with CNCM [21]	17.0	32.5	16.0	49.32	196.8
Baseline with StripConv	16.1	30.4	14.3	16.45	45.5
Baseline with LSKA	15.1	29.2	13.3	13.86	25.6
Baseline with LKA	16.7	31.8	15.4	15.54	26.4
Baseline with SDBConv	16.8	32.2	15.5	20.54	62.4
Baseline with LREA	17.2	32.7	16.4	21.55	68.8

Overall, different neck enhancement modules exhibit substantial differences in the trade-off between detection accuracy and computational efficiency. Lightweight modules such as RGCSPELAN, FCM, FMB, and InceptionNeXt Block reduce the number of parameters or GFLOPs, but their performance improvements are limited. For example, RGCSPELAN reduces the computational cost to 33.9 GFLOPs but decreases AP from 16.4% to 15.5%, while InceptionNeXt Block also results in lower AP and AP_50_ than the baseline. This suggests that excessive lightweight design may weaken fine-grained feature representation for UAV tiny-object detection. Existing large-kernel or directional context modules also show different behaviors. StripConv and LSKA reduce computational complexity to 45.5 and 25.6 GFLOPs, respectively, but their AP drops to 16.1% and 15.1%, indicating that simply introducing strip convolution or separable large-kernel attention does not necessarily improve tiny-object perception. LKA achieves better efficiency with 26.4 GFLOPs and improves AP to 16.7%, but its AP_75_ remains lower than the baseline, suggesting that fixed large-kernel aggregation may be insufficient for precise localization. In contrast, SDBConv improves AP to 16.8% and AP_50_ to 32.2%, demonstrating that decomposed broad receptive-field modeling can provide more effective contextual representation than conventional strip or large-kernel designs. It is worth noting that CNCM achieves relatively high performance, with 17.0% AP, 32.5% AP_50_, and 16.0% AP_75_. However, this improvement comes at a considerable computational cost, as its parameter count and complexity increase sharply to 49.32M and 196.8 GFLOPs, respectively. By comparison, the proposed LREA achieves the best overall performance on the CODrone dataset, reaching 17.2% AP, 32.7% AP_50_, and 16.4% AP_75_, while keeping the parameter count and computational cost at 21.55M and 68.8 GFLOPs. Compared with SDBConv alone, LREA further improves AP, AP_50_, and AP_75_, indicating that the BSA mechanism provides additional benefits by adaptively selecting complementary contextual responses across different branches. These results demonstrate that LREA achieves a more favorable balance between accuracy and efficiency, and its advantage comes not merely from enlarging the receptive field, but from combining decomposed broad receptive-field modeling with adaptive multi-scale and directional feature aggregation.

**Fig 12:**
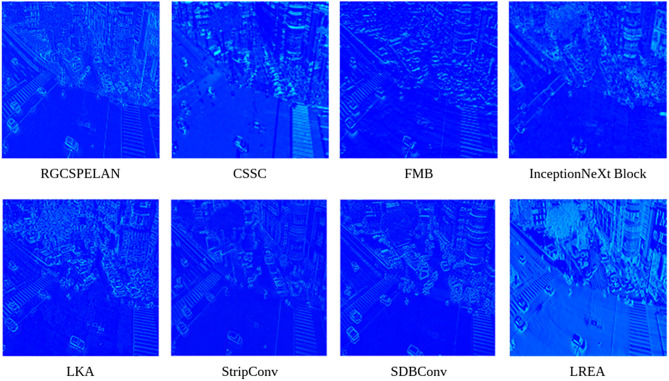
Feature maps processed by different feature enhancement modules.

To provide a more intuitive comparison of the impact of different neck modules on feature representation, Fig 12 presents the feature maps produced by several representative feature enhancement modules on the CODrone dataset, including RGCSPELAN, CSSC, FMB, InceptionNeXt Block, LKA, StripConv, SDBConv, and the proposed LREA. As shown in the figure, the model exhibits noticeable differences in target perception as the neck enhancement module changes. Compared with conventional lightweight enhancement modules, large-kernel or strip-convolution-based modules can capture broader contextual information, but their feature responses are still relatively scattered or affected by background textures. SDBConv strengthens the responses around object regions by introducing decomposed broad receptive-field modeling. Furthermore, when the full LREA is adopted, the resulting feature maps show clearer target contours and richer local details. This advantage is especially evident for tiny objects, where LREA not only expands the receptive field but also adaptively integrates multi-scale and directional contextual information through BSA, thereby enhancing the discriminability of target features.

### Training stability analysis

**Fig 13:**
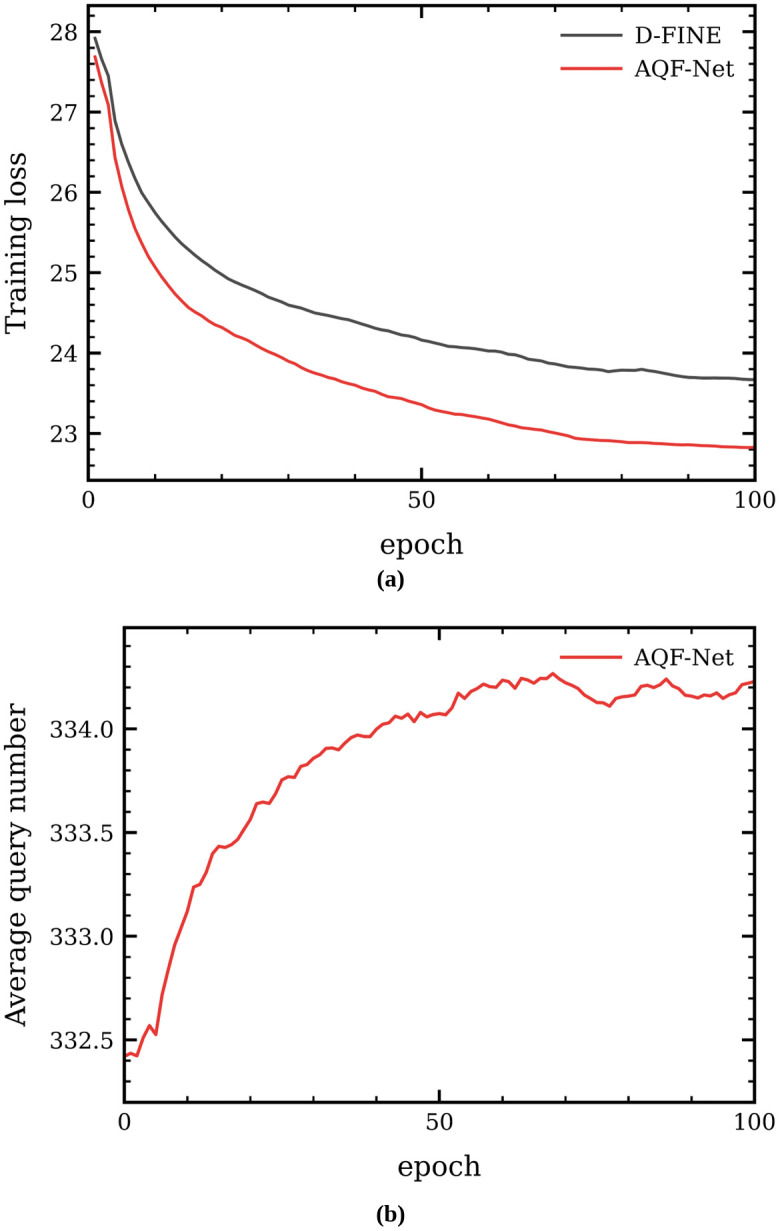
Training stability analysis of AQF-Net. (a) Training loss curves of D-FINE and AQF-Net. (b) Average query number evolution of AQF-Net during training.

To further analyze whether the adaptive query modeling strategy affects training stability, we visualize the training loss curves and the average query number evolution, as shown in Fig 13.

Fig 13(a) shows that both D-FINE and AQF-Net exhibit smooth and stable convergence under the same training schedule. Compared with the D-FINE baseline, AQF-Net achieves a consistently lower training loss and converges to a lower final loss value, indicating that the proposed modules do not introduce optimization instability. Fig 13(b) further illustrates the evolution of the average number of decoder queries during training. The average query number increases gradually in the early training stage and then becomes stable in the later stage, with only small fluctuations. This suggests that the Categorical Counting Module provides stable density-aware guidance for query allocation rather than causing abrupt query oscillations. Overall, these results demonstrate that AQF-Net maintains stable convergence behavior while dynamically adjusting the query budget according to scene density.

### Visualization analysis

#### D-FINE-M AQF-Net-M.

**Fig 14:**
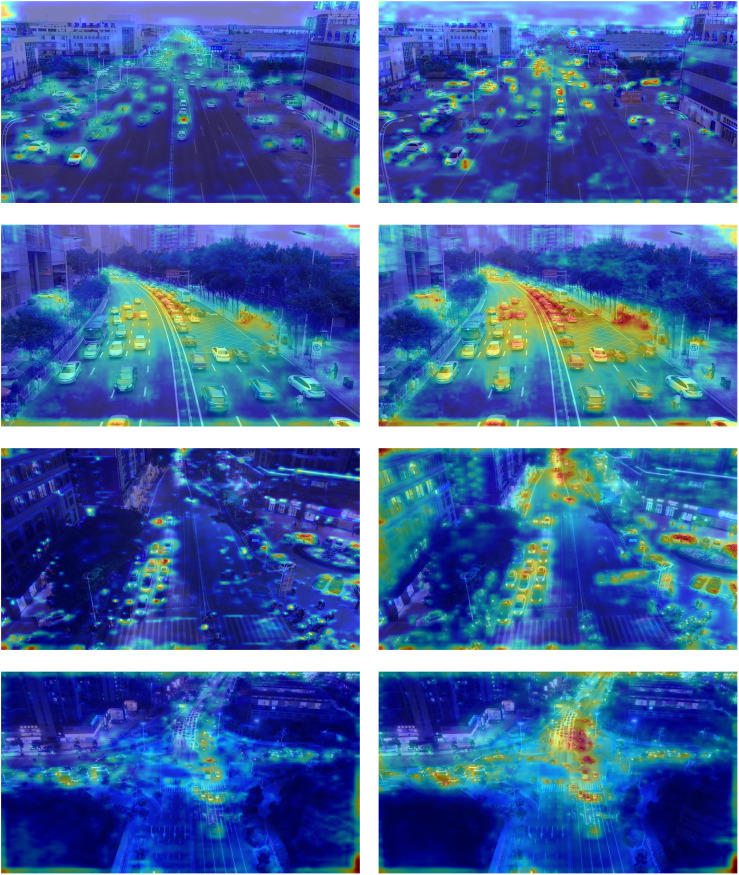
Heatmap comparison between D-FINE-M and AQF-Net-M.

Fig 14 presents a comparison of the feature response heatmaps of D-FINE-M and AQF-Net-M on representative samples from the VisDrone and CODrone datasets. It can be observed that, under complex background conditions, the activation regions of D-FINE-M are relatively scattered, and its responses to some tiny objects are not sufficiently prominent, making the model more susceptible to interference from background textures and noise. In contrast, AQF-Net-M produces more concentrated and clearer high-response distributions in object regions. Especially in scenarios with densely distributed tiny-scale objects, the model is able not only to generate high-confidence activations at target locations, but also to effectively suppress responses in irrelevant background regions. These results indicate that, after incorporating LREA and FQSA, the model improves its multi-scale contextual modeling capability while maintaining computational efficiency, thereby enabling it to focus more consistently on tiny-object regions. In addition, the adaptive query modeling strategy, through counting-guided feature enhancement and query selection, allows the model to adaptively adjust its attention regions in scenarios with large variations in object density, resulting in more discriminative response distributions in the heatmaps.

### Detection comparison

#### D-FINE-M AQF-Net-M.

**Fig 15:**
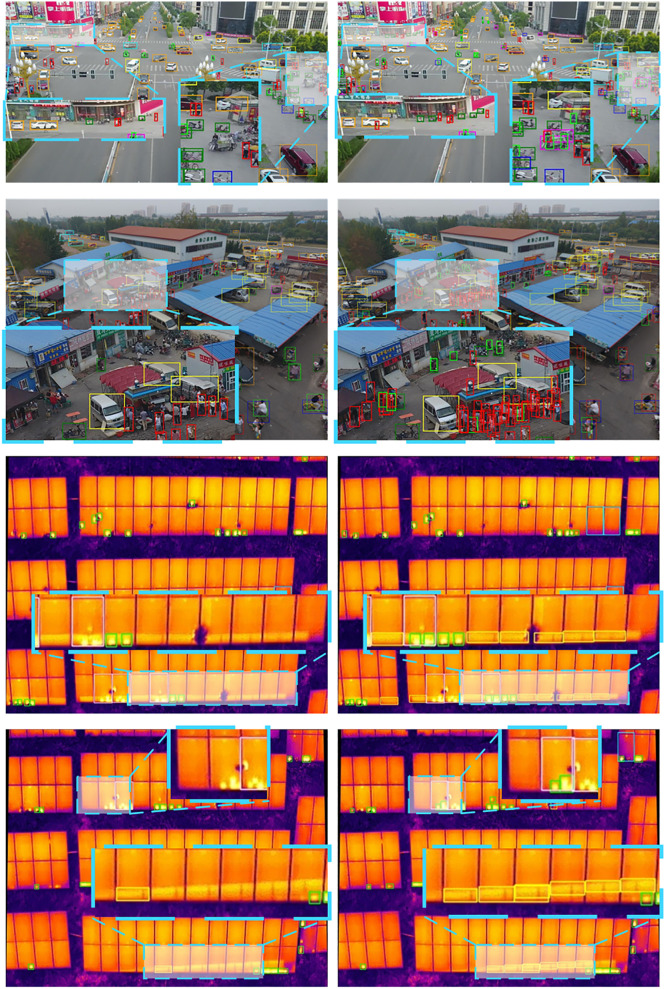
Detection results comparison on VisDrone and PV-DV datasets.

Fig 15 presents a visual comparison of the detection results of D-FINE-M and AQF-Net-M on the VisDrone and PV-DV datasets. As can be observed, in the VisDrone scenario, AQF-Net-M is able to detect more densely distributed tiny objects while significantly reducing both missed detections and false positives. On the PV-DV dataset for photovoltaic defect detection, AQF-Net-M not only maintains a high recall rate but also achieves more accurate defect localization results. This advantage can be mainly attributed to the ability of AQF-Net-M to adaptively adjust the number of queries according to object density, thereby improving detection stability and robustness in different scenarios.

## Discussion

Overall, the proposed AQF-Net achieves a favorable balance between detection accuracy and computational efficiency across diverse UAV aerial scenarios. By integrating efficient attention modeling, enhanced multi-scale feature representation, and adaptive query allocation, the framework demonstrates consistent performance improvements on multiple benchmarks and strong robustness in practical industrial applications such as photovoltaic defect detection. Nevertheless, the current experiments should not be interpreted as a complete validation of cross-domain generalization. In real UAV applications, the data distribution may vary substantially due to changes in flight altitude, imaging angle, illumination condition, weather, sensor type, compression quality, and scene layout. Although the results on CODrone, VisDrone2019, and PV-DV indicate that AQF-Net is effective across multiple UAV-related datasets, more strictly controlled cross-domain evaluations are still needed to fully assess its transferability under unseen acquisition conditions.

**Fig 16:**
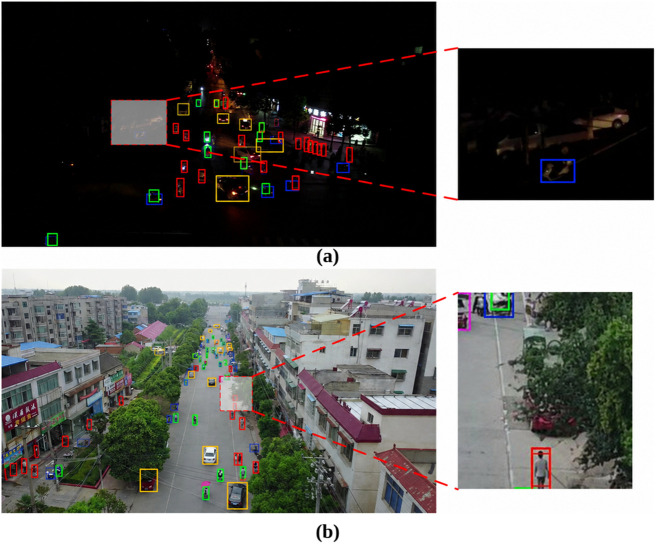
Representative failure cases of AQF-Net in challenging UAV scenarios. (a) Nighttime or low-light scene, where weak illumination reduces the contrast between tiny objects and the background. (b) Tree/building occlusion scene, where object boundaries are partially obscured and may lead to missed detections or inaccurate localization.

Despite the overall effectiveness of AQF-Net, several challenging cases remain. As shown in Fig 16, detection performance may degrade under nighttime or low-light conditions, where weak illumination reduces the contrast between tiny objects and the background. In addition, partial occlusion caused by trees, buildings, or other infrastructure may obscure object boundaries and lead to missed detections or inaccurate localization. These cases indicate that UAV tiny-object detection is still sensitive to severe illumination changes, occlusion, and domain shifts caused by complex real-world acquisition environments. In future work, we will further investigate illumination-robust feature enhancement, occlusion-aware modeling, cross-altitude and cross-weather evaluation, cross-sensor transferability, and multi-frame temporal information to improve detection robustness in more complex real-world UAV scenarios.

## Conclusion

To address the challenges of limited detection accuracy, frequent missed detections, and false positives caused by extremely small object scales, dense distributions, complex backgrounds, and large variations in object numbers in UAV aerial imagery, we propose AQF-Net, a UAV tiny-object detection framework built upon D-FINE. The proposed method systematically improves the original framework in terms of attention computation efficiency, feature enhancement, and query adaptability, thereby enhancing detection performance and stability in complex aerial scenarios. The main contributions and conclusions of this work are summarized as follows:

(1) A Fixed-Query Self-Attention (FQSA) mechanism is proposed. By performing self-attention on a fixed number of low-resolution query tokens, FQSA effectively reduces the computational complexity under high-resolution features. Meanwhile, it preserves global contextual modeling capability and improves computational efficiency in high-resolution UAV aerial scenarios.
(2) A Large-Receptive-Field Enhancement (LREA) module is designed. By integrating the Sparse Decomposed Broad Convolution (SDBConv) module with the Bidirectional Selective Aggregation (BSA) module, LREA achieves receptive field expansion and adaptive multi-scale feature fusion under relatively low computational overhead, thereby enhancing tiny-object representation and improving robustness in complex backgrounds.
(3) An adaptive query modeling strategy is introduced. Guided by counting-based dynamic query allocation, this strategy adaptively adjusts the number of queries according to object density in each image. As a result, it alleviates the performance bottleneck of fixed-query mechanisms in both sparse and dense object scenarios, while improving target matching quality and detection stability.

Experimental results demonstrate that AQF-Net achieves consistent performance improvements across multiple datasets. On the CODrone dataset, AQF-Net-M attains 17.8% AP and 34.4% AP_50_, representing improvements of 1.4% and 3.5% over D-FINE-M, respectively. On the VisDrone2019 validation set, AQF-Net-M achieves 33.4% AP and 55.0% AP_50_, outperforming the D-FINE-M baseline by 4.6% and 6.9%, respectively. On the PV-DV photovoltaic defect dataset, AQF-Net-M reaches 58.7% AP and 85.5% AP_50_, exceeding D-FINE-M by 1.8% and 1.7%, respectively, indicating superior defect detection accuracy and localization capability.

## References

[1] Zhou Y, Li J, Ou C, Yan D, Zhang H, Xue X. Open-Vocabulary Object Detection in UAV Imagery: A Review and Future Perspectives. Drones. 2025;9(8):557. doi: 10.3390/drones9080557

[2] Zheng J, Li L. GDEIM-SF: A Lightweight UAV Detection Framework Coupling Dehazing and Low-Light Enhancement. Sensors. 2026;26(5):1557. doi: 10.3390/s26051557 41829518 PMC12986597

[3] Bai Y, Liu Y. DL-DEIM: An Efficient and Lightweight Detection Framework with Enhanced Feature Fusion for UAV Object Detection. Applied Sciences. 2025;15(22):11966. doi: 10.3390/app152211966

[4] Ding J, Xue N, Xia G-S, Bai X, Yang W, Yang MY, et al. Object Detection in Aerial Images: A Large-Scale Benchmark and Challenges. IEEE Trans Pattern Anal Mach Intell. 2022;44(11):7778–96. doi: 10.1109/TPAMI.2021.3117983 34613910

[5] Liu M, Wang X, Zhou A, Fu X, Ma Y, Piao C. UAV-YOLO: Small Object Detection on Unmanned Aerial Vehicle Perspective. Sensors (Basel). 2020;20(8):2238. doi: 10.3390/s20082238 32326573 PMC7218847

[6] Wang J, Zhang F, Zhang Y, Liu Y, Cheng T. Lightweight Object Detection Algorithm for UAV Aerial Imagery. Sensors (Basel). 2023;23(13):5786. doi: 10.3390/s23135786 37447639 PMC10346989

[7] Wang G, Chen Y, An P, Hong H, Hu J, Huang T. UAV-YOLOv8: A Small-Object-Detection Model Based on Improved YOLOv8 for UAV Aerial Photography Scenarios. Sensors (Basel). 2023;23(16):7190. doi: 10.3390/s23167190 37631727 PMC10458807

[8] Qiu X, Chen Y, Cai W, Niu M, Li J. LD-YOLOv10: A Lightweight Target Detection Algorithm for Drone Scenarios Based on YOLOv10. Electronics. 2024;13(16):3269. doi: 10.3390/electronics13163269

[9] Vaswani A, Shazeer N, Parmar N, Uszkoreit J, Jones L, Gomez AN. Attention Is All You Need. Advances in Neural Information Processing Systems. 2017;30. doi: 10.48550/arXiv.1706.03762

[10] Li F, Zeng A, Liu S, Zhang H, Li H, Zhang L, et al. Lite DETR: An Interleaved Multi-Scale Encoder for Efficient DETR. In: 2023 IEEE/CVF Conference on Computer Vision and Pattern Recognition (CVPR). 2023;18558–67. doi: 10.1109/cvpr52729.2023.01780

[11] Zhang H, Li F, Liu S, Zhang L, Su H, Zhu J, et al. DINO: DETR with Improved DeNoising Anchor Boxes for End-to-End Object Detection. arXiv preprint. 2022. doi: 10.48550/arXiv.2203.03605

[12] Zhao Y, Lv W, Xu S, Wei J, Wang G, Dang Q, et al. DETRs Beat YOLOs on Real-time Object Detection. In: 2024 IEEE/CVF Conference on Computer Vision and Pattern Recognition (CVPR). 2024;16965–74. doi: 10.1109/cvpr52733.2024.01605

[13] Kong Y, Shang X, Jia S. Drone-DETR: Efficient Small Object Detection for Remote Sensing Image Using Enhanced RT-DETR Model. Sensors (Basel). 2024;24(17):5496. doi: 10.3390/s24175496 39275406 PMC11397902

[14] Peng Y, Li H, Wu P. D-FINE: Redefine regression task in DETRs as fine-grained distribution refinement. In: 2024. 10.48550/arXiv.2410.13842

[15] Zou S, Li C, Sun H, Xu P, Zhang J, Ma P, et al. TOD-CNN: An effective convolutional neural network for tiny object detection in sperm videos. Comput Biol Med. 2022;146:105543. doi: 10.1016/j.compbiomed.2022.105543 35483229

[16] Wang J, Huang Z, Dong Y, Hu Y, Zhao H. LE-YOLO: a lightweight and effective YOLO model for remote sensing image object detection. J Real-Time Image Proc. 2025;23(1). doi: 10.1007/s11554-025-01822-8

[17] Wu J, Pan Z, Lei B, Hu Y. FSANet: Feature-and-Spatial-Aligned Network for Tiny Object Detection in Remote Sensing Images. IEEE Trans Geosci Remote Sensing. 2022;60:1–17. doi: 10.1109/tgrs.2022.3205052

[18] Zhang Y, Xiao Y, Zhang Y, Zhang T. Video saliency prediction via single feature enhancement and temporal recurrence. Engineering Applications of Artificial Intelligence. 2025;160:111840. doi: 10.1016/j.engappai.2025.111840

[19] Jiang L, Yuan B, Du J, Chen B, Xie H, Tian J, et al. MFFSODNet: Multiscale Feature Fusion Small Object Detection Network for UAV Aerial Images. IEEE Trans Instrum Meas. 2024;73:1–14. doi: 10.1109/tim.2024.3381272

[20] Lv Z, Dong S, He J, Hu B, Liu Q, Wang H. Lightweight sewer pipe crack detection method based on amphibious robot and improved YOLOv8n. Sensors (Basel). 2024;24(18):6112. doi: 10.3390/s24186112 39338857 PMC11435957

[21] Yuan S, Qin H, Yan X, Yang S, Yang S, Akhtar N, et al. ASCNet: asymmetric sampling correction network for infrared image destriping. IEEE Trans Geosci Remote Sensing. 2025;63:1–15. doi: 10.1109/tgrs.2025.3534838

[22] Xu Z, Wu D, Yu C, Chu X, Sang N, Gao C. SCTNet: Single-Branch CNN with Transformer Semantic Information for Real-Time Segmentation. AAAI. 2024;38(6):6378–86. doi: 10.1609/aaai.v38i6.28457

[23] Xiao Y, Xu T, Xin Y, Li J. FBRT-YOLO: Faster and Better for Real-Time Aerial Image Detection. AAAI. 2025;39(8):8673–81. doi: 10.1609/aaai.v39i8.32937

[24] Zheng M, Sun L, Dong J, et al. SMFANet: a lightweight self-modulation feature aggregation network for efficient image super-resolution. In: European Conference on Computer Vision. Cham: Springer Nature Switzerland; 2024. pp. 359–75. doi: 10.1007/978-3-031-72973-7_21

[25] Yu W, Zhou P, Yan S, Wang X. InceptionNeXt: When Inception Meets ConvNeXt. In: 2024 IEEE/CVF Conference on Computer Vision and Pattern Recognition (CVPR), 2024. 5672–83. doi: 10.1109/cvpr52733.2024.00542

[26] Liu HI, Huang YX, Shuai HH, et al. DQ-DETR: DETR with dynamic query for tiny object detection. arXiv e-prints. 2024. doi: 10.48550/arXiv.2404.03507

[27] Fan X, Xing B, Wang X, Liu H, Yan C, Zhi P. SAR-D-FINE: A Context-Aware Detector for Small and Densely Packed Ship Detection in SAR Imagery. IEEE Geosci Remote Sensing Lett. 2026;23:1–5. doi: 10.1109/lgrs.2025.3640683

[28] Yu L, Tian Y, Chen J, Chi C, Li T, Li J. Balancing Accuracy and Speed: Improved D-FINE for Real-Time Ocean Internal Wave Detection. JMSE. 2026;14(4):388. doi: 10.3390/jmse14040388

[29] Hu Q, Wang L. HF-D-FINE: High-resolution features enhanced D-FINE for tiny object detection in UAV image. Image and Vision Computing. 2026;165:105834. doi: 10.1016/j.imavis.2025.105834

[30] Dosovitskiy A, Beyer L, Kolesnikov A. An image is worth 16x16 words: Transformers for image recognition at scale. arXiv preprint. 2020. doi: 10.48550/arXiv.2010.11929

[31] Wan Q, Huang Z, Lu J. SeaFormer: squeeze-enhanced axial Transformer for mobile semantic segmentation. 2023. doi: 10.48550/arXiv.2301.13156

[32] Liu S, Chen T, Chen X. More convnets in the 2020s: Scaling up kernels beyond 51x51 using sparsity. 2022. https://arxiv.org/abs/2207.03620

[33] Lau KW, Po LM, Rehman YAU. Large Separable Kernel Attention: Rethinking the Large Kernel Attention Design in CNN. Expert Systems with Applications. 2024;236:121352. doi: 10.1016/j.eswa.2023.121352

[34] Lin X, Yan Z, Deng X, et al. ConvFormer: plug-and-play CNN-style Transformers for improving medical image segmentation. In: International Conference on Medical Image Computing and Computer-Assisted Intervention. Cham: Springer Nature Switzerland; 2023. pp. 642–51. doi: 10.48550/arXiv.2309.05674

[35] Cai X, Lai Q, Wang Y, Wang W, Sun Z, Yao Y. Poly Kernel Inception Network for Remote Sensing Detection. In: 2024 IEEE/CVF Conference on Computer Vision and Pattern Recognition (CVPR), 2024. 27706–16. 10.1109/cvpr52733.2024.02617

[36] Ye K, Tang H, Liu B. More clear, more flexible, more precise: a comprehensive oriented object detection benchmark for UAV. arXiv preprint. 2025. doi: 10.48550/arXiv.2504.20032

[37] Cao Y, He Z, Wang L, Wang W, Yuan Y, Zhang D, et al. VisDrone-DET2021: The Vision Meets Drone Object detection Challenge Results. In: 2021 IEEE/CVF International Conference on Computer Vision Workshops (ICCVW). 2021. 2847–54. doi: 10.1109/iccvw54120.2021.00319

[38] Jocher G, Chaurasia A, Qiu J. Ultralytics YOLOv8 (Version 8.0.0). 2023. Available: https://github.com/ultralytics/ultralytics

[39] Wang CY, Yeh IH, Liao HYM. YOLOv9: learning what you want to learn using programmable gradient information. In: European Conference on Computer Vision. Cham: Springer Nature Switzerland; 2024. pp. 1–21. doi: 10.48550/arXiv.2402.13616

[40] Wang A, Chen H, Liu L, Chen K, Lin Z, Han J, et al. YOLOv10: Real-Time End-to-End Object Detection. In: Advances in Neural Information Processing Systems 37, 2024. 107984–8011. 10.52202/079017-3429

[41] Khanam R, Hussain M. YOLOv11: An Overview of the Key Architectural Enhancements. arXiv preprint. 2024. doi: 10.48550/arXiv.2410.17725

[42] Zhu X, Su W, Lu L. Deformable detr: Deformable transformers for end-to-end object detection. arXiv preprint. 2020. doi: 10.48550/arXiv:2010.04159

[43] Yang C, Huang Z, Wang N. QueryDet: Cascaded Sparse Query for Accelerating High-Resolution Small Object Detection. In: 2022 IEEE/CVF Conference on Computer Vision and Pattern Recognition (CVPR). 2022. 13658–67. doi: 10.1109/cvpr52688.2022.01330

[44] Zhang W, Hong Z, Xiong L, Zeng Z, Cai Z, Tan K. Sinextnet: A New Small Object Detection Model for Aerial Images Based on PP-Yoloe. Journal of Artificial Intelligence and Soft Computing Research. 2024;14(3):251–65. doi: 10.2478/jaiscr-2024-0014

[45] Liu C, Gao G, Huang Z, Hu Z, Liu Q, Wang Y. YOLC: You Only Look Clusters for Tiny Object Detection in Aerial Images. IEEE Trans Intell Transport Syst. 2024;25(10):13863–75. doi: 10.1109/tits.2024.3386928

[46] Du B, Huang Y, Chen J, Huang D. Adaptive Sparse Convolutional Networks with Global Context Enhancement for Faster Object Detection on Drone Images. In: 2023 IEEE/CVF Conference on Computer Vision and Pattern Recognition (CVPR). 2023;13435–44. doi: 10.1109/cvpr52729.2023.01291

[47] Wang P, Zhao J. Sod-Yolo: enhancing Yolo-based detection of small objects in UAV imagery. arXiv preprint. 2025. doi: 10.48550/arXiv.2507.12727

[48] Zhang H, Zhang H, Liu K, Gan Z, Zhu G-N. UAV-DETR: Efficient End-to-End Object Detection for Unmanned Aerial Vehicle Imagery. In: 2025 IEEE/RSJ International Conference on Intelligent Robots and Systems (IROS). 2025. 15143–9. doi: 10.1109/iros60139.2025.11246176

[49] Zhang H, Zhang H, Mei A, Gan Z, Zhu G-N. SO-DETR: Leveraging Dual-Domain Features and Knowledge Distillation for Small Object Detection. In: 2025 International Joint Conference on Neural Networks (IJCNN). 2025. 1–8. doi: 10.1109/ijcnn64981.2025.11229240

[50] Huang S, Lu Z, Cun X, Yu Y, Zhou X, Shen X. DEIM: DETR with Improved Matching for Fast Convergence. In: 2025 IEEE/CVF Conference on Computer Vision and Pattern Recognition (CVPR). 2025. 15162–71. doi: 10.1109/cvpr52734.2025.01412

